# Functional analysis of the sporulation-specific diadenylate cyclase CdaS in *Bacillus thuringiensis*

**DOI:** 10.3389/fmicb.2015.00908

**Published:** 2015-09-14

**Authors:** Cao Zheng, Yang Ma, Xun Wang, Yuqun Xie, Maria K. Ali, Jin He

**Affiliations:** ^1^State Key Laboratory of Agricultural Microbiology, College of Life Science and Technology, Huazhong Agricultural UniversityWuhan, China; ^2^Key Laboratory of Fermentation Engineering (Ministry of Education), College of Bioengineering, Hubei University of TechnologyWuhan, China

**Keywords:** cyclic di-AMP, *Bacillus thuringiensis*, CdaS, sporulation, parasporal crystal

## Abstract

Cyclic di-AMP (c-di-AMP) is a recently discovered bacterial secondary messenger molecule, which is associated with various physiological functions. In the genus *Bacillus*, the intracellular level and turnover of c-di-AMP are mainly regulated by three diadenylate cyclases (DACs), including DisA, CdaA and CdaS, and two c-di-AMP-specific phosphodiesterases (GdpP and PgpH). In this study, we demonstrated that CdaS protein from *B. thuringiensis* is a hexameric DAC protein that can convert ATP or ADP to c-di-AMP *in vitro* and the N-terminal YojJ domain is essential for the DAC activity. Based on the markerless gene knock-out method, we demonstrated that the transcription of *cdaS* was initiated by the sporulation-specific sigma factor σ^H^ and the deletion of *cdaS* significantly delayed sporulation and parasporal crystal formation. These findings contrast with similar experiments conducted using *B. subtilis*, wherein transcription of its *cdaS* was initiated by the sigma factor σ^G^. Deletion of all the three DAC genes from a single strain was unsuccessful, suggesting that c-di-AMP is an indispensable molecule in *B. thuringiensis*. Phylogenetic analysis indicated increased diversity of CdaS in the *B. cereus* and *B. subtilis Bacillus* subgroups. In summary, this study identifies important aspects in the regulation of c-di-AMP in the genus *Bacillus*.

## Introduction

Nucleotide second messengers are representative intracellular signaling molecules in all domains of life. In bacteria, cyclic AMP (cAMP) and tetra- or pentaphosphate [(p)ppGpp], the first two nucleotide molecules discovered, have been classically associated with carbon metabolism and the stringent response, respectively (Camilli and Bassler, 2006). In contrast to its complex and wide-ranging regulatory roles in eukaryotes, the involvement of cGMP in bacterial processes has only recently been investigated, where it mainly participates in the development of bacterial cysts and in bacterial phytopathogenesis (Gomelsky, 2011; Marden et al., 2011; An et al., 2013). Bacteria can also utilize cyclic dinucleotides such as c-di-GMP and c-di-AMP to respond to diverse types of stimuli (Corrigan and Gründling, 2013; Kalia et al., 2013). The signaling machinery of the c-di-GMP pathway, including synthetases, degrading enzymes, receptors and effectors have been studied intensively in many bacteria (Römling et al., 2013). Conversely, c-di-AMP is a recently identified messenger molecule and less is known for its metabolism and physiological functions. c-di-AMP has been reported as a sensor of DNA integrity in *B. subtilis* (Oppenheimer-Shaanan et al., 2011; Gándara and Alonso, 2015), to maintain cell wall homeostasis in several species (Corrigan et al., 2011; Luo and Helmann, 2012b; Witte et al., 2013), to help mediate survival of *Staphylococcus aureus* in low-potassium concentrations (Corrigan et al., 2013; Bai et al., 2014), and to regulate central metabolism (Sureka et al., 2014). Additionally, c-di-AMP secreted from some pathogens triggers a host type-I interferon and interferon-mediated signaling pathway (Woodward et al., 2010; Parvatiyar et al., 2012; Barker et al., 2013; Kaplan Zeevi et al., 2013), which plays an important role in infection and disease. Very recently, the hybrid cyclic nucleotide 3′5′-3′5′ cyclic GMP-AMP (cGAMP) was discovered in *Vibrio cholerae* and was shown to be implicated in intestinal colonization (Davies et al., 2012).

c-di-AMP was originally identified by Witte et al. (2008) as a bound product during the structural analysis of DNA integrity scanning protein (DisA) from *Thermotoga maritime*. They also reported that the DUF147 domain of DisA, as well as its homolog from *Bacillus subtilis*, converts two ATP molecules into one c-di-AMP molecule. Thus the DUF147 domain is also known as a diadenylate cyclase (DAC) domain. Furthermore, Römling (2008) found that the 120-amino acid-long DAC domain was distributed in nearly 300 species of bacteria and archaea. The DHH/DHHA1 domain of c-di-AMP-specific GdpPs (GGDEF domain protein-containing phosphodiesterases (PDE)) that degrades c-di-AMP to 5′-pApA, or further to 5′-AMP (Rao et al., 2010; Bai et al., 2013; Cho and Kang, 2013; Du et al., 2014; Ye et al., 2014), was also found in proteins from diverse bacteria. More recently, a His-Asp (HD) domain-containing protein named PgpH was found to specifically hydrolyze c-di-AMP to 5′-pApA (Gundlach et al., 2015b; Huynh et al., 2015). Thus, the two functionally opposing DAC and PDE enzymes regulate bacterial c-di-AMP turnover and maintain intracellular c-di-AMP levels under certain conditions. DAC, DHH/DHHA1 or HD domains are often found adjacent to different types of domains and the genes encoding DACs or PDEs are normally located in operons with genes involved in numerous biological activities. These arrangements imply that the c-di-AMP pool might be regulated by various extra- and intracellular stimuli through multiple pathways and c-di-AMP may play an important role in the control of various cellular processes (Corrigan and Gründling, 2013; Kalia et al., 2013).

As an important component of signaling pathways, c-di-AMP-specific receptors have been identified in many species. These include the TetR-family transcription factor DarR in *Mycobacterium smegmatis* (Zhang et al., 2013), three proteins associated with ion transport in *S. aureus* (potassium transport component KtrA, cation proton antiporter CpaA, and histidine kinase protein KdpD) (Corrigan et al., 2013), pyruvate carboxylase (PC) in *L. monocytogenes* (Sureka et al., 2014), PII-like signal transduction proteins in *S. aureus* (PstA) and *B. subtilis* (DarA) (Campeotto et al., 2015; Gundlach et al., 2015a; Müller et al., 2015), STING and DDX41 proteins in mammalian cells (Parvatiyar et al., 2012), and *ydaO* riboswitches in many diverse bacterial species (Nelson et al., 2013; Gao and Serganov, 2014; Jones and Ferré-D'Amaré, 2014; Ren and Patel, 2014).

Compared with the majority of bacterial species which encode only one DAC enzyme, members of the genus *Bacillus* generally encode three DAC domain-containing proteins: DisA, CdaA (previously named YbbP in the genus *Bacillus* or DacA in other genera) and CdaS (previously named YojJ in the genus *Bacillus* or DacB in others). The functions and activities of these three proteins have been investigated in the Gram-positive model bacterium *B. subtilis* (Mehne et al., 2013). The DAC domain of DisA is connected to a C-terminal DNA binding domain. Upon DNA damage, DisA pauses at the DNA double-strand breakage sites, resulting in conformational changes in the N-terminal DAC domain which inhibit DAC catalytic activity and cause a delay in sporulation (Witte et al., 2008). Additionally, DAC activity of the *M. smegmatis* DisA was negatively affected by the DNA repair protein RadA (Zhang and He, 2013). CdaA is a transmembrane protein composed of a N-terminal three-helix membrane-spanning domain and a C-terminal DAC domain. The activity of CdaA is stimulated by a protein-protein interaction with CdaR (Mehne et al., 2013). The third DAC enzyme, CdaS (cyclic di-AMP synthase for sporulation-specific), contains a N-terminal YojJ domain and a C-terminal DAC domain. It was reported that *cdaS* was most likely under the control of the late forespore-specific sigma factor σ^G^ (Nicolas et al., 2012; Mehne et al., 2013), and the DAC activity of CdaS was auto-inhibited by its N-terminus (Mehne et al., 2014). Furthermore, deletion of *cdaS* also affected spore germination (Mehne et al., 2014). However, the *in vitro* DAC activity and physiological function of CdaS are poorly understood.

*B. thuringiensis* is another representative strain in the genus *Bacillus*, where it is categorized within the *B. cereus* group, which is distinct from the *B. subtilis* group (Anderson et al., 2005). One of the most significant features of *B. thuringiensis* is the formation of parasporal crystals during sporulation, which are composed of insecticidal crystal proteins (ICPs). The ICPs often possess insecticidal or nematicidal properties, therefore *B. thuringiensis* has been extensively used in pest control (Schnepf et al., 1998). The mechanisms of sporulation and ICP formation in *B. thuringiensis* have been investigated for many years (Baum and Malvar, 1995; Chang et al., 2001; Gong et al., 2012; Wang et al., 2013b,c), but little information concerning the signaling pathways involved in sporulation and ICP formation has been reported. In the present study, we investigated the c-di-AMP mediated signaling pathways in *B. thuringiensis*. For this purpose, we focused on the biochemical properties and physiological roles of CdaS in *B. thuringiensis*.

## Materials and methods

### Bacterial strains and culture conditions

The bacterial strains and plasmids used in this work are listed in Table 1. *B. thuringiensis* BMB171 (Li et al., 2000a,b; He et al., 2010), an acrystalliferous mutant strain with a high transformation frequency, was used as the parent strain in this study. Unless otherwise specified, BMB171 and its mutants were cultured at 28°C in GYS medium (g/L: glucose, 1; yeast extract, 2; K_2_HPO_4_·3H_2_O, 0.655; (NH_4_)_2_SO_4_, 2; MgSO_4_·7H_2_O, 0.041; MnSO_4_·H_2_O, 0.0378; CaCl_2_, 0.08). When necessary, relevant antibiotics were added to the cultures at the following final concentrations: 50 μg/mL for kanamycin, 25 μg/mL for erythromycin, 300 μg/ml for spectinomycin, or 60 units for polymyxin. *Escherichia coli* DH5α was used for routine cloning, and *E. coli* BL21(DE3) was used for expression of recombinant proteins. The *E. coli* strains were grown in Lysogeny broth (LB) medium or LB agar plates at 37°C. The antibiotic concentrations used for *E. coli* were: 50 μg/mL for kanamycin, 100 μg/mL for ampicillin, or 100 μg/mL for spectinomycin.

**Table 1:** **Bacterial strains and plasmids used in this study**.

**Bacteria or plasmids**	**Relevant characteristics**	**Origins**
**BACTERIAL STRAINS**		
*E. coli* BL21(DE3)	Protein expression host; F^−^*omp*T *hsd*S_B_(r${}_{\text{B}}^{-}$m${}_{\text{B}}^{-}$) *dcm*(DE3) *gal*λ	Beijing TransGen Biotech Co., Ltd
*E. coli* DH5α	RecA1 endA1 gyrA96 thi hsdR17(r${}_{\text{k}}^{-}$ m${}_{\text{k}}^{+}$) relA1 supE44 Φ80ΔlacZΔM15Δ(lacZYA-argF)U169	Beijing TransGen Biotech Co., Ltd
BMB171	*B. thuringiensis* strain BMB171; an acrystalliferous mutant strain; high transformation frequency	Li et al., 2000a,b
BMB171-I-*cdaS*	The red single cross-over insertion strain for knock-out of *cdaS* in BMB171	This work
Δ*cdaS*	*cdaS* mutant of BMB171	This work
BMB171-I-*cdaA*	The red single cross-over insertion strain for knock-out of *cdaA* in BMB171	This work
Δ*cdaA*	*cdaA* mutant of BMB171	This work
BMB171-I-*disA*	The red single cross-over insertion strain for knock-out of *disA* in BMB171	This work
Δ*disA*	*disA* mutant of BMB171	This work
Δ*disA*-I-*cdaS*	The red single cross-over insertion strain for knock-out of *cdaS* in Δ*disA*	This work
Δ*disA*Δ*cdaS*	*disA* and *cdaS* double mutant of BMB171	This work
Δ*cdaA*-I-*cdaS*	The red single cross-over insertion strain for knock-out of *cdaS* in Δ*cdaA*	This work
Δ*cdaA*Δ*cdaS*	*cdaA* and *cdaS* double mutant of BMB171	This work
Δ*disA*-I-*cdaA*	The red single cross-over insertion strain for knock-out of *cdaA* in Δ*disA*	This work
BMB171-I-*sigH*	The red single cross-over insertion strain for knock-out of *sigH* in BMB171	This work
Δ*sigH*	*sigH* mutant of BMB171	This work
BMB171-I-*sigF*	The red single cross-over insertion strain for knock-out of *sigF* in BMB171	This work
Δ*sigF*	*sigF* mutant of BMB171	This work
BMB171-I-*sigE*	The red single cross-over insertion strain for knock-out of *sigE* in BMB171	This work
Δ*sigE*	*sigE* mutant of BMB171	This work
BMB171-*cry1Ac*	BMB171 containing plasmid pBMB43-304, which expressed Cry1Ac10 in BMB171	This work
Δ*cdaS-cry1Ac*	Δ*cdaS* containing plasmid pBMB43-304, which expressed Cry1Ac10 in Δ*cdaS*	This work
**PLASMIDS**		
pET28b(+)	T7 promoter expression vector, Km^R^	Novagen
pET28b-*cdaS*	*cdaS* in NcoI and XhoI sites of pET28b, used for expression of CdaS in BL21(DE3)	This work
pET28b-*cdaS*_70−201_	*cdaS*_70−201_in NcoI and XhoI sites of pET28b, used for expression of CdaS_70−201_ in BL21(DE3)	This work
pET28b-*cdaS*_28−201_	*cdaS*_28−201_in NcoI and XhoI sites of pET28b, used for expression of CdaS_28−201_ in BL21(DE3)	This work
pET28b-*cdaS*_17−201_	*cdaS*_17−201_in NcoI and XhoI sites of pET28b, used for expression of CdaS_17−201_ in BL21(DE3)	This work
pET28b-*cdaS*_11−201_	*cdaS*_11−201_in NcoI and XhoI sites of pET28b, used for expression of CdaS_11−201_ in BL21(DE3)	This work
pET28b-*cdaS*_10−201_	*cdaS*_10−201_in NcoI and XhoI sites of pET28b, used for expression of CdaS_10−201_ in BL21(DE3)	This work
pET28b-*cdaS*_9−201_	*cdaS*_9−201_in NcoI and XhoI sites of pET28b, used for expression of CdaS_9−201_ in BL21(DE3)	This work
pET28b-*cdaS*_8−201_	*cdaS*_8−201_in NcoI and XhoI sites of pET28b, used for expression of CdaS_8−201_ in BL21(DE3)	This work
pET28b-*cdaS*_7−201_	*cdaS*_7−201_in NcoI and XhoI sites of pET28b, used for expression of CdaS_7−201_ in BL21(DE3)	This work
pET28b-*cdaS*_5−201_	*cdaS*_5−201_in NcoI and XhoI sites of pET28b, used for expression of CdaS_5−201_ in BL21(DE3)	This work
pET28b-*cdaS*_4−201_	*cdaS*_4−201_in NcoI and XhoI sites of pET28b, used for expression of CdaS_4−201_ in BL21(DE3)	This work
pET28b-*cdaS*_3−201_	*cdaS*_3−201_in NcoI and XhoI sites of pET28b, used for expression of CdaS_3−201_ in BL21(DE3)	This work
pET28b-*cdaS*_*W*4*G*_	*cdaS*_*W*4*G*_ in NcoI and XhoI sites of pET28b, used for expression of CdaS_W4G_ in BL21(DE3)	This work
pET28b-*cdaS*_*W*4*A*_	*cdaS*_*W*4*A*_ in NcoI and XhoI sites of pET28b, used for expression of CdaS_W4A_ in BL21(DE3)	This work
pET28b-*cdaS*_*W*4*D*_	*cdaS*_*W*4*D*_ in NcoI and XhoI sites of pET28b, used for expression of CdaS_W4D_ in BL21(DE3)	This work
pET28b-*cdaS*_*W*4*K*_	*cdaS*_*W*4*K*_ in NcoI and XhoI sites of pET28b, used for expression of CdaS_W4K_ in BL21(DE3)	This work
pET28b-*cdaS*_*DGA*_	*cdaS*_*DGA*_ in NcoI and XhoI sites of pET28b, used for expression of CdaS_DGA_ in BL21(DE3)	This work
pET28b-*cdaS*_*RHR*_	*cdaS*_*RHR*_ in NcoI and XhoI sites of pET28b, used for expression of CdaS_RHR_ in BL21(DE3)	This work
pET28b-*cdaS*_*DGA*∕*RHR*_	*cdaS*_*DGA*∕*RHR*_ in NcoI and XhoI sites of pET28b, used for expression of CdaS_DGA∕RHR_ in BL21(DE3)	This work
pET28b-*cdaA*_86−273_	*cdaA*_86−273_ in NcoI and XhoI sites of pET28b, used for expression of CdaA_86−273_ in BL21(DE3)	This work
pHT1K	*B. thuringiensis*-*E. coli* shuttle plasmid; Amp^R^ Erm^R^	Kang et al., 2005; Liao et al., 2014
pHT1K-*lacZ*	pHT1K vector harboring the promoterless *lacZ* gene, transformed into BMB171 and used for β-galactosidase activity	Wang et al., 2013a
pHT1K-*P_*cdaS*_*-*lacZ*	*lacZ* with the promoter of *cdaS* in NcoI and BglII sites of pHT1K, transformed into BMB171 and used for β-galactosidase activity assay	This work
pHT1K-*P_*cdaA*_*-*lacZ*	*lacZ* with the promoter of *cdaA* in NcoI and BamHI sites of pHT1K, transformed into BMB171 and used for β-galactosidase activity assay	This work
pHT1K-*P_*disA*_*-*lacZ*	*lacZ* with the promoter of *disA* in NcoI and BamHI sites of pHT1K, transformed into BMB171 and used for β-galactosidase activity assay	This work
pHT1K-*P_*gdpP*_*-*lacZ*	*lacZ* with the promoter of *gdpP* in NcoI and BamHI sites of pHT1K, transformed into BMB171 and used for β-galactosidase activity assay	This work
pBMB43-304	*B. thuringiensis-E. coli* shuttle plasmid containing ORF of *cry1Ac10*; Amp^R^ Erm^R^	Qi et al., 2015
pRP1028	*B. thuringiensis*-*E. coli* shuttle plasmid; Amp^R^ Erm^R^; containing *turbo-rfp* gene and an I-SceI recognition site	Janes and Stibitz, 2006
pSS4332	*B. thuringiensis*-*E. coli* shuttle plasmid; Km^R^; containing *gfp* and I-SceI restriction enzyme encoding gene	Janes and Stibitz, 2006
pSS1827	The helper plasmid for conjugative transfer; Amp^R^	Janes and Stibitz, 2006
pRP1028-*cdaS*UD	pRP1028 with the upstream and downstream regions of *cdaS*, intermediate vector in gene-knockout experiments	This work
pRP1028-*cdaA*UD	pRP1028 with the upstream and downstream regions of *cdaA*, intermediate vector in gene-knockout experiments	This work
pRP1028-*disA*UD	pRP1028 with the upstream and downstream regions of *disA*, intermediate vector in gene-knockout experiments	This work
pRP1028-*sigH*UD	pRP1028 with the upstream and downstream regions of *sigH*, intermediate vector in gene-knockout experiments	This work
pRP1028-*sigF*UD	pRP1028 with the upstream and downstream regions of *sigF*, intermediate vector in gene-knockout experiments	This work
pRP1028-*sigE*UD	pRP1028 with the upstream and downstream regions of *sigE*, intermediate vector in gene-knockout experiments	This work
pMD19-T simple vector	Intermediate cloning vector	Takara

### Protein purification

PCR primer pairs used in subcloning are listed in Table S1. All the PCR products were digested and inserted into the Novagen pET-28b(+) vector using 5′ NcoI and 3′ XhoI restriction sites (underlined). Recombinant plasmids (Table 1) containing the correct sequences were then transformed into *E. coli* BL21(DE3) competent cells to obtain the corresponding expression strains.

Protein expression and Ni-NTA affinity purification were performed following a previously described procedure (Zheng et al., 2013). All the proteins used in this study are C-terminal His-tagged proteins, for which the results of purification are shown as SDS-PAGE images in Figure S1.

The full-length CdaS protein eluted from a Ni-NTA affinity column was filtered through a 0.45 μm syringe filter, and then applied to a Source-Q 5-100 column which was connected to an AKTA Fast Protein Liquid Chromatography (FPLC) system (GE Healthcare, Piscataway, NJ, USA). Elution was achieved with a linear gradient from 0 to 1 M NaCl in equilibration buffer (25 mM Tris-HCl, pH 8.0) at a flow rate of 1.5 mL/min from 0 to 50 min. Protein elution was monitored by UV detection (280 nm), and target fractions were collected and pooled.

For gel filtration chromatography experiments, the pooled CdaS protein was concentrated to 1 mL (approximately 10 mg/mL) using ultrafiltration and loaded on a Superdex 200HR 10/30 column (GE Healthcare, Piscataway, NJ, USA) that was pre-equilibrated and eluted with lysis buffer (25 mM Tris-HCl, pH 8.0, 150 mM NaCl) at a flow rate of 0.5 mL/min using a Bio-Rad FPLC system (Bio-Rad, Richmond, CA, USA). The Superdex 200HR 10/30 column was adjusted using a mix of protein standards (Bio-Rad) consisting of thymoglobin (670 kDa), γ-globulin (158 kDa), ovalbumin (44 kDa), myoglobin (17 kDa) and vitamin B12 (1.35 kDa).

### Analytical ultracentrifugation

Sedimentation velocity (SV) experiments were performed on a Beckman-Coulter ProteomeLab XL-A analytical ultracentrifuge (Beckman-Coulter, Fullerton, CA, USA) using double sector or six-channel centerpieces and sapphirine windows (Wang et al., 2012). Before the experiment, the wild type CdaS protein was dialyzed in 25 mM Tris-HCl (pH 8.0) buffer, and its OD_280_ was adjusted to 1.0. The SV experiment was conducted at 50 000 rpm. The buffer parameters (density and viscosity) and protein partial specific volume (V-bar) were obtained using SEDNTERP software. The SV data was analyzed by the *c*(*M*) method as part of the SEDFIT program.

### Enzymatic activity assays

An initial reaction mixture containing 100 mM HEPES at pH 7.5, 10 mM MgCl_2_, and 200 μM nucleotides in a total volume of 100 μL was used to detect the enzymatic activity of CdaS. The reaction was initiated by adding 1 μM protein, and the reaction mixture was incubated at 37°C for 2 h. The reaction was then terminated by heating the reaction mixture in a boiling water bath for 5 min, and the mixture was centrifuged at 16,000 × g for 10 min to remove the denatured protein. Subsequently, 10 μL of the supernatant was loaded onto a SHIMADZU Prominence Modular high performance liquid chromatography (HPLC) system (Shimadzu Corporation, Kyoto, Japan), which contains a LC-20AT liquid chromatograph, a SHL-20A Auto Sampler, a CTO-20A column oven (temperature was maintained at 25°C), and a G1314C UV/VIS detector (wavelength was set at 254 nm), to monitor the nucleotides. The separation of nucleotides was achieved on an Agela Technologies Innoval C18 column (250 × 4.6 mm; 5 μm particle diameter) and the gradient elution program was *t* = 0–20 min, 86% A, 14% B; *t* = 20–22 min, 86–75% A, 14–25% B; *t* = 22–32 min, 75% A, 25% B; *t* = 32–35 min, 75–86% A, 25–14% B; *t* = 35–50 min, 86% A, 14% B. Solvent A was 100 mM KH_2_PO_4_ and 4 mM tetrabutylammonium bromide (pH was adjusted to 6.0 using KOH), solvent B was methanol. The acquisition time was 35 min and the flow rate was set at 1 mL/min throughout the program.

The assay conditions used for pH screening were: 10 mM MgCl_2_, 200 μM ATP, 1 μM CdaS, and 100 mM MES (pH 5.5–6.5), 100 mM HEPES (pH 7.0–8.0), and 100 mM CHES (pH 8.6–10.0), respectively. For metal screening, 10 mM Mg^2+^ was replaced with 10 mM of another divalent metal cation (Mn^2+^, Ca^2+^, Ba^2+^, Sr^2+^, Zn^2+^, Cu^2+^, or Ni^2+^) in the initial conditions. For Mg^2+^ concentration screening, experiments were conducted by adding MgCl_2_ to the reaction mixture at various final concentrations over a range from 0 to 100 mM.

### LC/Q-TOF analysis of c-di-AMP

Liquid chromatography/quadrupole time-of-flight tandem mass spectrometry (LC/Q-TOF) analysis was performed on an Agilent 1260 LC system [consisting of a G1322A degasser, a G1312B binary pump, a G1367E thermostated autosampler, G1316A thermostated column compartment, and a G4212B diode array detector (DAD)] (Agilent Technologies, Santa Clara, CA, USA) coupled to an ultra high definition quadrupole time-of-flight mass spectrometer Model 6540 (Agilent Technologies, Santa Clara, CA, USA) equipped with a dual source electrospray ionization ion source. The sample treatment, LC separation and Q-TOF parameters were conducted according to previously described methods (Tang et al., 2015).

The analytes were separated on an Agilent C18 reverse-phase column (100 × 1.8 mm, particle size of 3.5 μm). The binary mobile phase composed of 2% methanol and 98% water (containing 0.2% ammonium acetate and 0.1% acetic acid) was set at a constant flow rate of 300 μL/min and the column temperature was kept at 30°C, and the sample volume was 2 μL. Q-TOF parameters were as previously reported (Tang et al., 2015).

### Gene knock-out procedure for *B. thuringiensis*

The gene knock-out system in *B. thuringiensis* was developed based on homing endonuclease I-SceI mediated markerless gene replacement method established for *B. anthracis* by Janes and Stibitz (2006). For example, to construct the *cdaS* deletion mutant Δ*cdaS*, approximately 1000 bp of the upstream and downstream sequences flanking *cdaS* gene were amplified by PCR. These two fragments were digested and ligated into the temperature-sensitive shuttle plasmid pRP1028, resulting in the integrating plasmid pRP1028-*cdaS*UD, and the fidelity of inserted fragments were verified by sequencing and enzyme digestion. pRP1028 includes an I-SceI recognition site for I-SceI restriction endonuclease cleavage and one oriT site for conjugative transfer. The vector also encodes RFP as a reporter protein and possesses a spectinomycin resistance marker for convenient screening. The subsequent procedures are illustrated in Figure S2A.

### Construction of transcriptional fusion plasmids

The promoter-5′-UTR of *cdaS, cdaA, gdpP*, and *radA* (*radA* locates in the upstream region of *disA* within the same operon) genes were amplified using primer pairs listed in Table S1. The PCR products were digested with corresponding restriction endonucleases and ligated into the *lacZ*-containing shuttle plasimd pHT1K-*lacZ*, which we previously constructed (Wang et al., 2013a). Constructs were transformed into *E. coli* DH5α to acquire the plasmids pHT1K-*P*_*cdaS*_-*lacZ*, pHT1K-*P*_*cdaA*_-*lacZ*, pHT1K-*P*_*gdpP*_-*lacZ*, and pHT1K-*P*_*disA*_-*lacZ*, respectively (Table 1).

After confirmation by sequencing, the plasmids were extracted from *E. coli* DH5α and transformed (electroporation) into BMB171. All transformants were obtained by screening the clones in LB plates with 25 μg/mL erythromycin.

### Determination of β-galactosidase activity

BMB171 containing transcriptional fusion plasmids pHT1K-*P*_*cdaS*_-*lacZ*, pHT1K-*P*_*cdaA*_-*lacZ*, pHT1K-*P*_*gdpP*_-*lacZ*, and pHT1K-*P*_*disA*_-*lacZ* (Table 1) were grown at 28°C in an orbital shaker at 200 rpm in 200 mL GYS medium with 25 μg/mL erythromycin. 4 mL of cultures were collected at 2 h intervals and used for the determination of β-galactosidase activity (Wang et al., 2013a).

### RNA extraction, cDNA synthesis and RT-PCR

Twenty mL of a sample cultured for 18 h in GYS medium was centrifuged, and cell pellets were ground in liquid nitrogen to isolate total RNA using TRIzol reagent (Invitrogen, Carlsbad, CA). The final total RNA was analyzed by 1% agarose gel electrophoresis and quantified by NanoDrop (Thermo Scientific, USA). The PrimeScript™ RT reagent Kit with gDNA Eraser (TaKaRa, Japan) was used to synthesize cDNA according to the manufacturer's instructions. The final cDNAs were diluted and served as templates for PCR amplification of *cdaS* using specific primers (Table S1). The PCR products were separated on 1% agarose gel electrophoresis.

### Extraction and determination of Cry1Ac10 protein

Shuttle plasmid pBMB43-304 containing the ORF of *cry1Ac10* was purified from *E. coli* DH5α and transformed (electroporation) into BMB171 and Δ*cdaS* to produce BMB171-*cry1Ac* and Δ*cdaS*-*cry1Ac* strains, respectively. BMB171-*cry1Ac* and Δ*cdaS*-*cry1Ac* were grown in GYS medium at 28°C and 200 rpm. At the indicated time point (19 h), 20 mL of each culture was separately collected. The procedure for the extraction of Cry1Ac10 protein was previously reported (Wang et al., 2013b). Finally, concentration of Cry1Ac10 protein was measured by Bradford method and purity was analyzed by SDS-PAGE.

### Quantification of intracellular c-di-AMP concentration by reversed-phase LC-MS/MS

Different DAC mutant strains were cultured at 28°C for 18 h at 200 rpm, then cells were harvested (200 mL cultures) immediately by centrifugation at 4°C, and the cell pellets were extracted immediately using the nucleotide extraction method reported by Burhenne and Kaever (2013). Detection of c-di-AMP was performed on a Finnigan Surveyor Plus liquid chromatography system followed by a Thermo Scientific TSQ Quantum Ultra EMR tandem mass spectrum system (San Jose, CA, USA). Intracellular c-di-AMP level was normalized by the corresponding total protein concentration (Tang et al., 2015).

## Results

### Oligomerization of CdaS from *B. thuringiensis* BMB171

**Figure 1:**
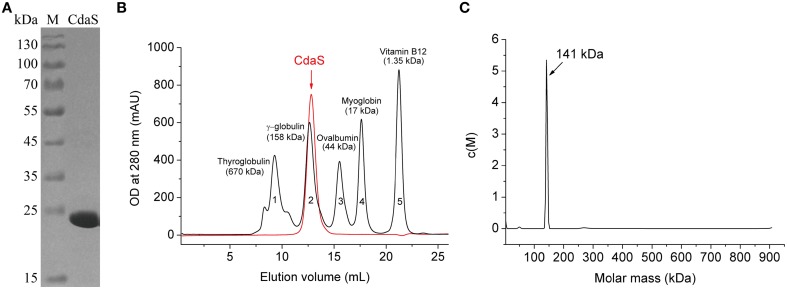
**Purification and oligomerization of *B. thuringiensis* CdaS**. **(A)** 12% SDS-PAGE analysis of purified CdaS. Lane M is the PageRulerTM Prestained Protein Ladder Marker (Thermo Scientific). **(B)** Superdex 200 Gel filtration chromatography of CdaS. **(C)** Analytical ultracentrifugation with sedimentation velocity of CdaS. Molecular mass of CdaS was estimated by SEDFIT software using the *c*(*M*) method.

To explore the oligomerization of *B. thuringiensis* CdaS, the recombinant His-tagged CdaS was purified from *E. coli* BL21(DE3) harboring pET28b-*cdaS* (Table 1) using Ni-NTA agarose chromatography. As shown by SDS-PAGE (Figure 1A), CdaS has an approximate molecular mass of 22 kDa, which is consistent with its monomer theoretical value (23.3 kDa). In principle, DAC proteins usually exist in multimeric forms in their natural state (Witte et al., 2008; Mehne et al., 2014). Gel filtration chromatography showed the purified CdaS, which was eluted at the elution volume (indicated with an arrow) close to 158 kDa (γ-globulin) (Figure 1B), suggesting that CdaS forms a hexamer (~141 kDa). For further confirmation, analytical ultracentrifugation with sedimentation velocity analysis of CdaS exhibited a narrow sedimentation coefficient distribution in continuous size distribution analysis (Figure 1C), demonstrating that it was mono-disperse stable hexamer. Taken together, *B. thuringiensis* CdaS, like its homologs from *B. cereus* (PDB code 2FB5) and *B. subtilis* (Mehne et al., 2014) exists as a hexamer in solution.

### *B. thuringiensis* CdaS is a DAC that can use ATP or ADP as a substrate

We examined the enzymatic properties of *B. thuringiensis* CdaS by determining its enzymatic reaction products using HPLC. In order to achieve high resolution on a C18 column, an ion-pair agent was selected as the mobile phase to adequately separate nucleosides throughout this study.

**Figure 2:**
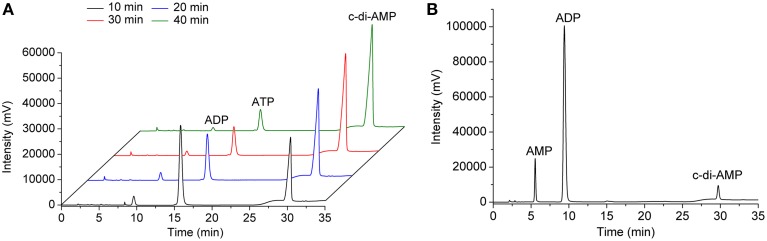
**Determination of the DAC activity of CdaS**. **(A)** Using ATP as a substrate. 1 μM CdaS was incubated with a standard reaction mixture (100 mM HEPES buffer (pH 7.5) containing 200 μM ATP and 10 mM MgCl_2_) at 37°C in a 500 μL reaction volume. 100 μL aliquots of the reaction were analyzed at different time points by HPLC. Black line: 10 min; blue line: 20 min; red line: 30 min; green line: 40 min. **(B)** Using ADP as a substrate. 1 μM CdaS was incubated with a standard reaction mixture (except that 200 μM ADP was substituted for ATP) at 37°C in 100 μL reaction volume for 8 h and then analyzed by HPLC.

The purified *B. thuringiensis* CdaS synthesized c-di-AMP from ATP. As shown in Figure 2A, a major product was eluted with the same retention time (~30 min) as was previously reported in *B. thuringiensis* DisA experiment (Zheng et al., 2013) as well as the commercial c-di-AMP standard (Figure S3A). The molecular mass of the major product measured by LC/Q-TOF was 659.1120 Da, which is identical to the theoretical formula weight of c-di-AMP (Figures S3B–D). Therefore, *B. thuringiensis* CdaS did indeed convert ATP into c-di-AMP, providing compelling evidence that it is a DAC enzyme.

In addition to the c-di-AMP chromatographic peak, a minor product peak (retention time 9.5 min) was present in the chromatograms. The retention time in chromatograms combined with the LC/Q-TOF results demonstrated that this peak represented ADP, raising the interesting possibility that ADP was the intermediate of c-di-AMP biosynthesis. To test this hypothesis, we monitored the enzymatic reaction process from 10 to 40 min. As shown in Figure 2A, the concentrations of both the substrate (ATP) and intermediate (ADP) decreased as the reaction progressed, while the concentration of c-di-AMP increased concomitantly. In theory, if ADP is an intermediate for the formation of c-di-AMP, CdaS can use ADP as a substrate to synthesize c-di-AMP directly. Therefore, we determined the enzymatic activity of CdaS with ADP as substrate. As expected, ADP was partially converted to c-di-AMP, but the yield of c-di-AMP was lower compared with that using ATP as the substrate (Figure 2B).

The effect of pH on the DAC activity of CdaS was measured at a wide range of buffer pH (5.5-10.0). Figure S4A shows that CdaS had relatively higher activities in alkaline conditions and pH 7.5 in HEPES buffer was determined to be the optimum reaction condition among the variables tested.

It has been reported that divalent cations are indispensable for the DAC activity of DisA (Witte et al., 2008; Bai et al., 2012; Rosenberg et al., 2015). Our results showed that the DAC activity of CdaS was strictly dependent on divalent cations (Figure S4B). Maximum enzymatic activity was found when Mg^2+^ was provided as a cofactor at a concentration of 10 mM (Figure S4C).

### The N-terminal region of *B. thuringiensis* CdaS is essential for the DAC activity

**Figure 3:**
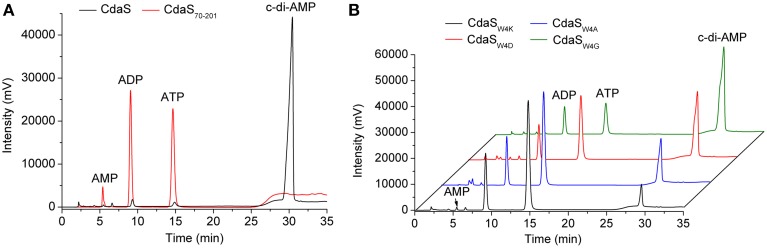
**The importance of the N-terminal region for the DAC activity of CdaS**. **(A)** 1 μM CdaS and CdaS_70−201_ were incubated with standard reaction mixture at 37°C in a 100 μL reaction volume for 8 h. **(B)** 1 μM of mutants CdaS_W4G_, CdaS_W4D_, CdaS_W4A_, and CdaS_W4K_ were reacted as indicated in **(A)** and analyzed by HPLC.

In many species, the DAC domain is fused with other domains in DAC proteins (Figure S5A), and some experimental evidence indicates that those domains are required for DAC activity (Bai et al., 2012; Rosenberg et al., 2015). Generally, CdaS contains a N-terminal YojJ domain in addition to the C-terminal DAC domain (http://pfam.xfam.org/). To test whether the YojJ domain of *B. thuringiensis* CdaS is important for DAC activity, we removed the YojJ domain by engineering a truncated CdaS (CdaS_70−201_), and then tested the DAC activity of this mutant protein. No c-di-AMP was detected despite of the presence of CdaS_70−201_ and abundant reaction time (overnight) (Figure 3A). This result suggested that the N-terminal YojJ domain of CdaS was required for its DAC activity.

To further examine the functional domains of CdaS, we generated a series of N-terminal truncated CdaS proteins CdaS_28−201_, CdaS_17−201_, CdaS_11−201_, CdaS_10−201_, CdaS_9−201_, CdaS_8−201_, CdaS_7−201_, CdaS_5−201_ and CdaS_4−201_(Figure S1). We found that CdaS_28−201_, CdaS_17−201_, and CdaS_11−201_ abolished DAC activity and CdaS_10−201_, CdaS_9−201_, CdaS_8−201_, CdaS_7−201_, and CdaS_5−201_ had only weak DAC activity, while CdaS_4−201_ was comparable with wild type CdaS (data not shown). These results indicate the N-terminal region from the fourth amino acid (Trp) to the ninth amino acid (Glu) represent key residues for CdaS's DAC activity (Figure S5B). To confirm this finding, the fourth amino acid Trp, the strongest nonpolar amino acid, was mutated into other types of amino acids, such as the weak nonpolar amino acid (Ala), uncharged polar amino acid (Gly), and positively (Lys) and negatively (Asp) charged polar amino acids. The DAC activities of these four mutant proteins decreased to different extents compared with the wild-type CdaS (Figure 3B) such that activity of CdaS_W4G_ > CdaS_W4D_ > CdaS_W4A_ > CdaS_W4K_.

Additionally, we expressed the soluble *B. thuringiensis* CdaA protein containing a deletion of the N-terminal transmembrane domain (CdaA_86−273_) (Figure S5A) and found that CdaA_86−273_ was unable to synthesize c-di-AMP (Figure S6), suggesting that the N-terminal transmembrane domain was essential for the DAC activity of CdaA.

### CdaS harbors weak ADPase activity

An ADP peak and a minor AMP peak co-existed in the chromatograms of enzymatic reaction products of all truncated and point-mutated CdaS (Figure 3). The AMP peak was observed when ADP was used as a substrate (Figure 2B). However, CdaS was unable to generate c-di-AMP when AMP was provided as a substrate. This demonstrated that CdaS converted ADP to AMP and possessed weak ADPase activity. This phenomenon was also observed in the reaction of *M. tuberculosis* DisA (Bai et al., 2012). However, the AMP peak was absent in reaction products catalyzed by wild-type CdaS with ATP as a substrate. A reasonable explanation is that the DAC activity is much stronger than ADPase activity for wild-type CdaS, converting most of the ATP to c-di-AMP through intermediate ADP. In contrast, the truncated and point-mutated CdaS proteins dramatically decreased or abolished DAC activities completely, thus revealing the ADPase activity.

**Figure 4:**
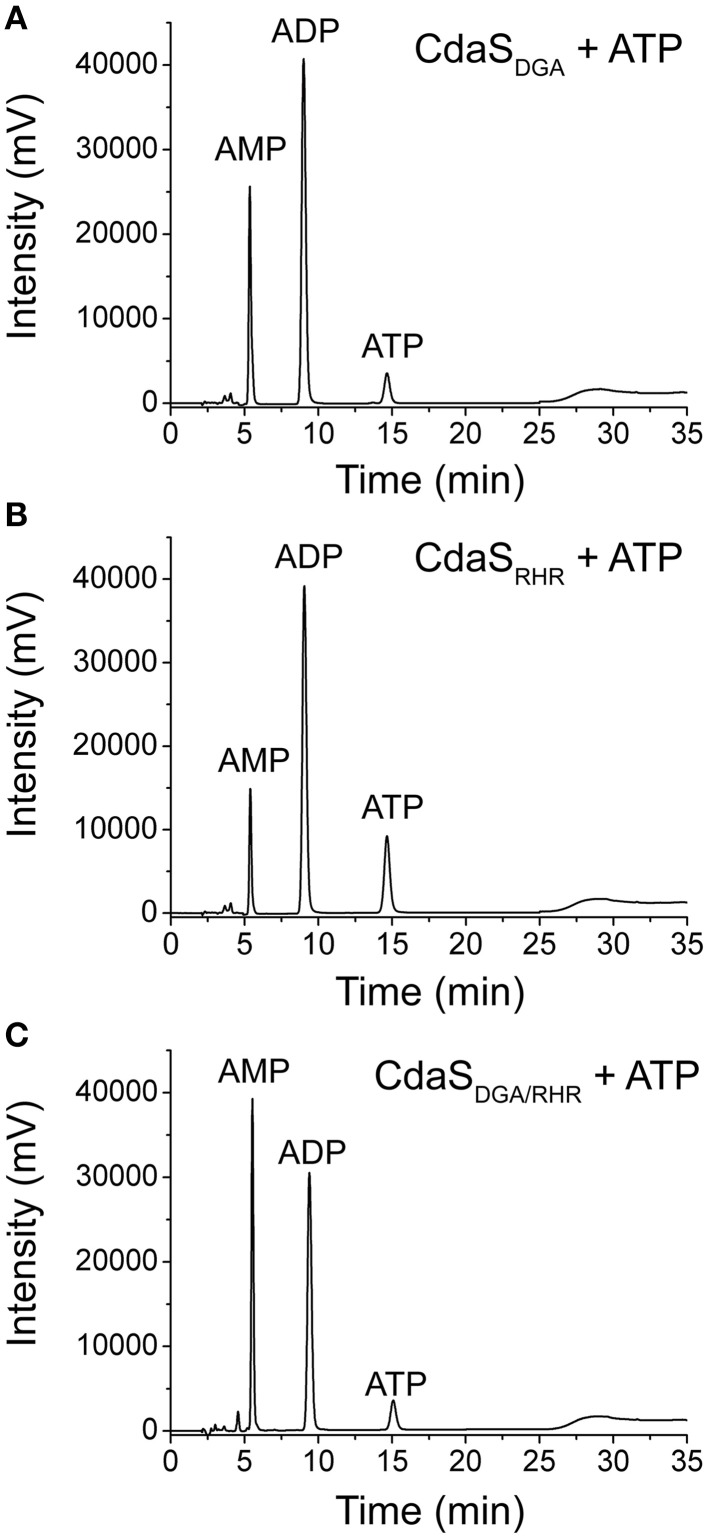
**Effect of DGA (A) and RHR (B) motifs alone and both motifs (C) on the DAC and ADPase activities of CdaS**. 1 μM of the three CdaS mutants (CdaS_DGA_, CdaS_RHR_, and CdaS_DGA∕RHR_) were individually incubated with standard reaction mixture at 37°C in a 100 μL reaction volume for 8 h and analyzed by HPLC.

All DAC proteins studied share two conserved functional motifs (DGA and RHR) for DAC activity (Witte et al., 2008; Bai et al., 2012). We constructed CdaS_DGA_ and CdaS_RHR_ mutants with either the individual DGA or RHR motifs mutated to AAA, as well as a CdaS_DGA∕RHR_ mutant with both motifs mutated to AAA. Figure 4 shows that the two single mutants and the double mutant completely abolished the formation of c-di-AMP, but still produced AMP, suggesting that DGA and RHR motifs were essential for CdaS's DAC activity but not for ADPase activity. Results demonstrated that the ADPase activity could be attributed to other active sites which are still unknown. Surprisingly, the double mutation resulted in increased ADPase activity compared with the two single mutants and the most likely explanation was that DGA and RHR might be the ATP-binding cassettes (Bai et al., 2012), and more ATP molecules were released from the double mutant to participate in the formation of AMP.

### Validation of the markerless gene deletion strains

A schematic diagram of the I-SceI mediated markerless gene knock-out method is provided (Figure S2A) and the detailed steps used to create mutations in *B. thuringiensis* are described in Materials and Methods Section. Here we present an example for PCR validation of the deletion of *cdaS*. In Step 2, the integrating plasmid pRP1028-*cdaS*UD (Table 1) was integrated into the chromosome of BMB171. We designed two pairs of primers (Table S1) and selected two red colonies (positive colonies) for PCR verification. One primer pair was located in upstream (CU-F) and downstream (CD-R) regions of *cdaS* gene. The electrophoretogram (Figure S2B) shows two PCR products, and the difference between the size of the larger product (1309 bp) and the smaller product (703 bp) is the length of *cdaS* (606 bp). The second primer pair used was CU-F and 1028-R (a reverse primer in pRP1028 vector), which yielded one distinct ~1700 bp PCR product (Figure S2B), further confirming the integration of pRP1028-*cdaS*UD.

In Step 4, six colonies exhibiting spectinomycin (300 μg/mL) sensitivity and kanamycin resistance were selected to amplify *cdaS* or its upstream and downstream regions using the CU-F and CD-R primer pair. If a mutant reverts to the parental strain, the size of PCR fragments should be 1309 bp (see lanes 1, 2, 3, and 5 in Figure S2B) which is identical to the positive control (BMB171 genomic DNA template). In the case that the *cdaS* gene is deleted, PCR fragments would be detected as 703 bp (lanes 4 and 6 in Figure S2B). The positive mutants were further confirmed by DNA sequencing (Figure S7) (The sequencing results for other mutants are showed in Figure S8–S12). Moreover, another primer pair C-F and C-R (the specific primers for *cdaS* gene) (Table S1) was used for independent validation. As expected, *cdaS* gene was amplified as shown in lanes 1, 2, 3, and 5 in Figure S2B, while a PCR product of the corresponding size was absent in lanes 4 and 6 in Figure S2B. Therefore two (colonies 4 and 6) out of six colonies were *cdaS* deletion strains.

### *CdaS* is transcribed in stationary phase

**Figure 5:**
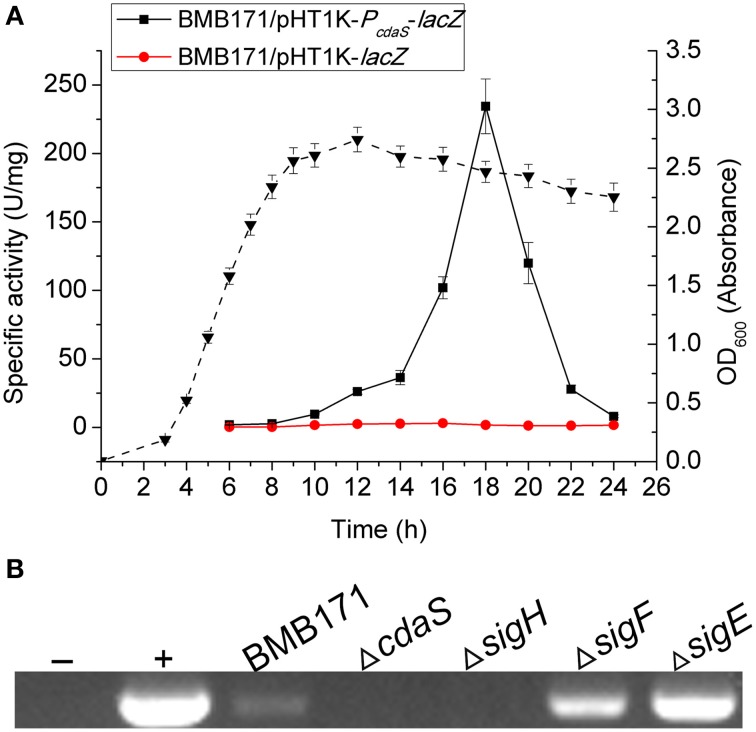
**The transcription of *cdaS***. **(A)** β-galactosidase activity of BMB171/pHT1K-*P*_*cdaS*_-*lacZ*. Strains were cultured in GYS medium at 28°C, and BMB171/pHT1K-*lacZ* was used as the negative control strain. The dashed line represents the growth curve of BMB171/pHT1K-*lacZ*. Data represent the mean of three independent experiments (error bar: standard error of the mean, SEM). **(B)** Detection of *cdaS* transcripts in different strains by RT-PCR. Total RNA was extracted from each sample after 18 h of growth in GYS medium. Lane -: H_2_O template; lane +: BMB171 genomic DNA template.

During sporulation, σ^H^ is considered to be the first active sigma factor regulating transcription in the predivisional cell. Four immediate sporulation-specific sigma factors σ^F^, σ^E^, σ^G^, and σ^K^ control transcription in the early forespore, early mother cell, late forespore, and late mother cell, respectively (de Hoon et al., 2010). A previous report demonstrated that *cdaS* was transcribed under the control of σ^G^ in *B. subtilis* (Nicolas et al., 2012; Mehne et al., 2013). To examine whether the transcription of *cdaS* is also sporulation-specific in *B. thuringiensis*, a pHT1K-*P*_*cdaS*_*-lacZ* plasmid encoding the *lacZ* reporter under the control of the *cdaS* promoter region was constructed and electroporated into BMB171 (Table 1). *cdaS* transcription was first detectable in the early-stationary phase (10 h, onset of sporulation), and attained its maximum value at 18 h (mid-stationary phase) (Figure 5A). Hence, *B. thuringiensis* CdaS expression is specifically regulated during sporulation and *cdaS* might be controlled by σ^H^, σ^F^, or σ^E^. To test this hypothesis, we used RT-PCR to examine *cdaS* transcription in different sigma factor mutants Δ*sigH*, Δ*sigF*, and Δ*sigE*. The results showed that *cdaS* was transcribed in BMB171 (as a positive control), Δ*sigF* and Δ*sigE*, but not in Δ*cdaS* (as a negative control) or in Δ*sigH* (Figure 5B). Therefore we determined that transcription of *B. thuringiensis cdaS* is σ^H^-dependent.

### CdaS promotes sporulation in *B. thuringiensis*

**Figure 6:**
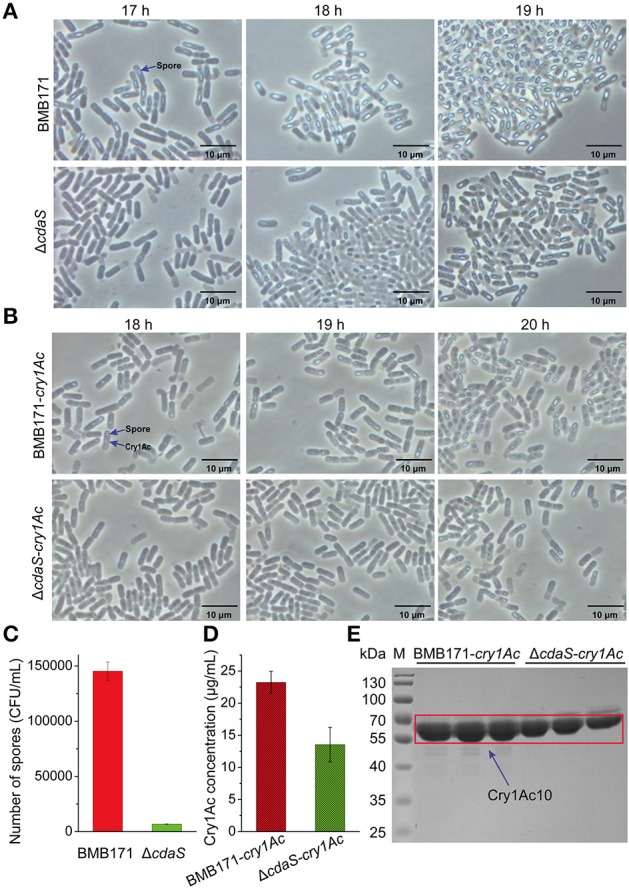
**Effect of *cdaS* deletion mutation on sporulation and parasporal crystal formation**. Time courses of the sporulation in BMB171 vs. Δ*cdaS* **(A)** and parasporal crystal formation in BMB171-*cry1Ac* vs. Δ*cdaS*-*cry1Ac* **(B)**. Strains were grown in GYS medium at 28°C and 200 rpm. Spore formation and parasporal crystal formation were monitored by phase-contrast microscopy at the indicated times. To test spore formation, 1 mL GYS culture samples at 18 h were heated at 80°C for 10 min, diluted in sterile water, and tested for colony formation on LB plates **(C)**. The concentrations of Cry1Ac10 at 19 h were measured by the Bradford method **(D)** and analyzed by SDS-PAGE **(E)**. Data are represented as the mean of three independent experiments (error bar: SEM). A two-tailed *t*-test was used for statistical analysis.

As its expression is sporulation-specific, CdaS might participate in the regulation of sporulation in *B. thuringiensis*. The growth curves obtained for BMB171 and Δ*cdaS* showed no significant differences (Figure S13). However, their spore formation rates were different. As shown in Figure 6A, approximately 50% of BMB171 spores were bright at 17 h in GYS medium, but the vast majority of Δ*cdaS* spores were still in phase dark. With the extension of incubation time, more spores turned from phase dark to phase bright in Δ*cdaS*, while the BMB171 spores became brighter. At 19 h, the number of spores with phase bright in Δ*cdaS* was equal to that of BMB171 at 17 h, indicating the spore formation in the mutant was delayed about 2 h. Similarly, the delay of parasporal crystal formation was synchronized with sporulation in Δ*cdaS*-*cry1Ac* compared with BMB171-*cry1Ac* (Figure 6B). Consistent with phase-contrast microscopy results, BMB171 produced 21 times more heat-resistant spores than Δ*cdaS* did at 18 h (Figure 6C). The measured Cry1Ac10 content in Δ*cdaS*-*cry1Ac* was significantly lower than that of BMB171-*cry1Ac* at 19 h (a two-tailed t-test was used for statistical analysis) (Figures 6D,E). Therefore, CdaS promotes both sporulation and the parasporal crystal formation in *B. thuringiensis*.

### c-di-AMP is an essential second messenger in *B. thuringiensis*

**Figure 7:**
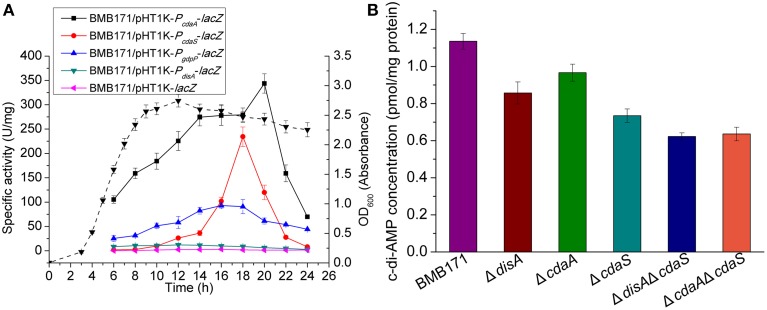
**(A)** Transcription of genes encoding c-di-AMP metabolic enzymes. Strains were cultured in GYS medium at 28°C and BMB/pHT1K-*lacZ* was used as the negative control. β-galactosidase assays measuring the activities of c-di-AMP related gene promoters were measured. The dashed line represents the growth curve of BMB171/pHT1K-*lacZ*. **(B)** Intracellular levels of c-di-AMP. Strains were cultured in GYS medium at 28°C for 18 h and extraction and measurement of c-di-AMP was performed as described in Materials and Methods Section. Data are represented as the mean of three independent experiments (error bar: SEM).

The distribution of genes (*disA, cdaA, cdaS, gdpP* and *pgpH*) that encode c-di-AMP related metabolic enzymes was analyzed in 78 strains of the genus *Bacillus* with complete genome sequences deposited at NCBI (Table S2). We found that all of the strains contain at least one of the three DAC genes and both the c-di-AMP-specific PDE genes. In *B. subtilis*, c-di-AMP is an important signaling molecule for cell viability (Mehne et al., 2013). To examine the biological function of c-di-AMP in *B. thuringiensis*, in more detail, we attempted to delete the DAC genes. Three single DAC gene mutants ▵*disA*, ▵*cdaA*, and ▵*cdaS*, and two double mutants ▵*disA*▵*cdaS* and ▵*cdaA*▵*cdaS* were constructed. However, we were unable to acquire the double mutant ▵*disA*▵*cdaA* and the triple mutant, suggesting that c-di-AMP is an essential molecule, and *disA* and *cdaA* could not be deleted simultaneously. Figure 7A showed that transcription of *disA* and *cdaA*, as well as that of *gdpP* started in exponential phase, whereas *cdaS* was transcribed only in stationary phase. If *disA* and *cdaA* are absent simultaneously, we reason that the bacterial cell would not be able to synthesize c-di-AMP in the initial growth stage and it will finally result in bacterial death. In this regard, c-di-AMP seems to be required for the viability of *B. thuringiensis*.

These mutants were studied by LC-MS/MS assays for the detection of intracellular c-di-AMP levels. As shown in Figure 7B, when compared with BMB171, the c-di-AMP concentrations in the three single mutants (▵*disA*, ▵*cdaA*, and ▵*cdaS*) were significantly lower. The ▵*cdaS* mutant exhibited the greatest decrease in c-di-AMP, suggesting that CdaS is the major enzyme determining the c-di-AMP pool at the sporulation stage. The c-di-AMP levels were further reduced in the two double mutants (▵*disA*▵*cdaS* and ▵*cdaA*▵*cdaS*). These mutants showed similar growth rates as the parent strain BMB171 in our study. Taken together, these results suggest that *B. thuringiensis* may have a sophisticated regulatory mechanism that maintains c-di-AMP at a regulated, moderate level to avoid growth restriction.

## Discussion

### A high efficiency gene knock-out method was developed in *B. thuringiensis*

In addition to its use as an environmentally compatible biopesticide (Schnepf et al., 1998), *B. thuringiensis* has many advantages as a bacterial model, such as rapid growth and high plasmid stability. BMB171 also exhibits a particularly high transformation efficiency (Li et al., 2000a). These unique features enable BMB171 to be used not only as a stable expression system for gene function studies, but also as a production platform for the synthesis of a variety of different chemicals (Wang et al., 2013a). Thus, an alternative gene knock-out method with superior efficiency is a valuable tool for research involving *B. thuringiensis*.

In this study, a gene knock-out procedure was developed for *B. thuringiensis* based on a markerless gene replacement method stimulated by double-strand-breaks in the chromosome. Compared with classical chromosomal modification based on the insertion of a drug resistance selectable marker, the markerless gene knock-out method edits target genes without alerting other parts of the chromosome. During the process of gene knock-out, the pRP1028 derivative was able to replicate in BMB171, which enhanced the first recombination efficiency. At the same time, compared with the other plasmid system (pMAD) that was used in the *B. thuringiensis* HD-73 strain for gene deletion (Yang et al., 2012), RFP protein expressed from pRP1028 derivative improves screening efficiency, and growth at routine temperature (37°C) completely removes nomadic temperature-sensitive pRP1028 derivatives. More significantly, the double-strand breaks generated by the I-SceI cleavage stimulate the second recombination in this deletion procedure. Based on dozens of genes deletion in BMB171, we demonstrated the convenience of this system and the fact that it allows for the clean deletion of more than one gene within an operon as well as multiple deletions of different genes (using a single deletion mutant as the parent strain for the deletion of the next gene of interest, and so on).

### CdaS is a verified DAC in *B. thuringiensis*

In the present study, we have observed that CdaS from *B. thuringiensis* functions as a DAC and exhibits residual ADPase activity. These findings are consistent with a previous study on the *M*. *tuberculosis* DisA enzyme by Bai et al. (2012). They observed the formation of pApA during the enzyme catalytic reaction with ATP or ADP as a substrate. Analogously, a recent study revealed that *M*. *tuberculosis* DisA synthesized c-di-AMP through an intermediate pppApA (Manikandan et al., 2014). Nevertheless, we did not find either pApA or pppApA in CdaS catalytic reactions using high resolution mass spectrometry (Q-TOF), which indicated that the mechanism of c-di-AMP synthesis in *B. thuringiensis* CdaS is distinct from that of *M*. *tuberculosis* DisA.

### The N-terminal region is critical for DAC activity and the multimerization of CdaS

Several lines of evidence indicate that peptide domains apart from the DAC domain influence the oligomeric state and enzymatic activity of DAC proteins (Mehne et al., 2014; Bai et al., 2012). We identified the N-terminal of *B. thuringiensis* CdaS to be indispensable for DAC activity and confirmed that *B. thuringiensis* CdaS formed a hexamer, which was in accordance with results obtained for its analogs in *B. cereus* (PDB code 2FB5) and *B. subtilis* (Mehne et al., 2014). More importantly, we found that in addition to the two conserved functional motifs (DGA and RHR), the N-terminal region from the fourth amino acid (Trp) to the ninth amino acid (Glu) played a major role in the DAC activity of CdaS. Mutations in one of the residues (N-terminal fourth amino acid, Trp, W) significantly reduced the ability of CdaS to synthesize c-di-AMP. The amino acid sequence of *B. thuringiensis* CdaS is identical to that of *B. cereus* CdaS, and the crystal structure of *B. cereus* CdaS (PDB code 2FB5) showed that it oligomerizes as a trimer, pentamer and hexamer. The hexamer likely forms through two trimers *via* the interaction of C-terminal DAC domains, and c-di-AMP was expected to be synthesized in the cavity of two interacting DAC domains (Witte et al., 2008). We speculated that three monomers initially attract each other *via* electrostatic interaction, and then tightly bind through hydrophobic interactions (Figure S14). Trp is the most nonpolar amino acid and it is likely that the N-terminal fourth amino acid (Trp, W) is the major contributor to hydrophobic interactions for trimerization (Figure S14). Thus, when we deleted the N-terminal YojJ domain or replaced the N-terminal fourth amino acid (Trp, W) by other amino acids, the hydrophobic interactions may be decreased significantly. This may have reduced the possibility for trimerization, and further for hexamerization, weakening or abolishing the DAC activity.

### CdaS diversity in the genus *Bacillus*

A recent study suggested that the N-terminal domain serves to limit the enzymatic activity of the *B. subtilis* CdaS DAC domain (Mehne et al., 2014). However our result showed that the N-terminal domain was absolutely necessary for its DAC activity. The similarity of the two CdaS proteins in N-terminal YojJ domains is less than 20% with respect to amino acid sequence (Figure S5), although the overall protein similarity is 51%, indicating that the N-terminal conformational folds could be largely different from each other, and ultimately lead to the reversed regulatory mechanism.

**Figure 8:**
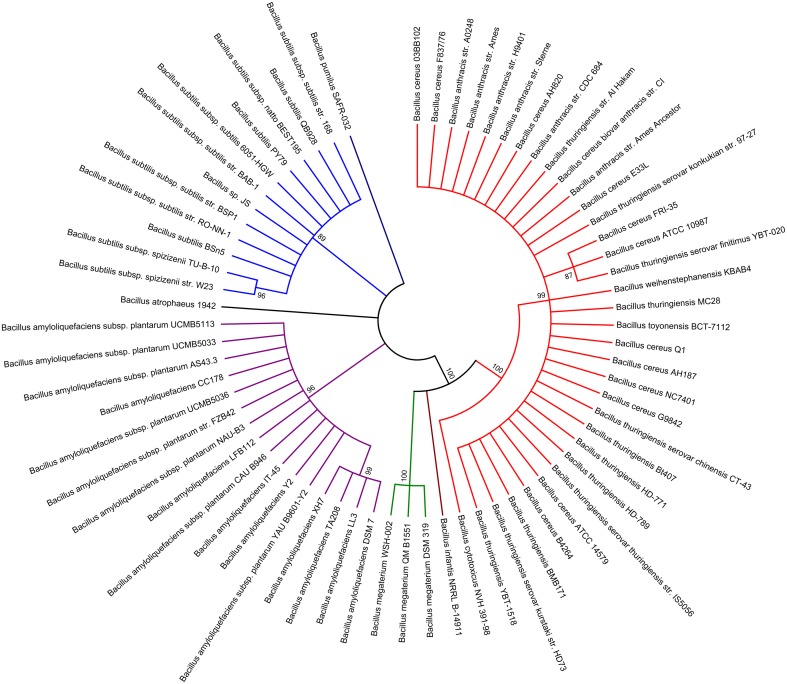
**Phylogenetic distribution of the CdaS proteins in the genus *Bacillus***. The analysis involved 68 CdaS amino acid sequences from each of 68 strains of the genus *Bacillus* with available genome reference sequences. The individual groups are demarcated with different colors, red: *B. cereus* group, Group I; brown: *B. infantis*, Group II; green: *B. megaterium*, Group III; purple: *B. amyloliquefaciens*, Group IV; dark: *B. atrophaeus*; Group V; blue: *B. subtilis*, Group VI, dark blue: *B. pumilus*, Group VII.

In order to further investigate the genetic diversity of CdaS from different species in the genus *Bacillus*, a phylogenetic tree of CdaS proteins from 68 strains of the genus *Bacillus* was constructed using MEGA5 software and the maximum likelihood method (Figure 8). On the whole, all CdaS proteins could be assembled into seven distinct groups. CdaS proteins from the *B. cereus* group, including *B. anthracis, B. thuringiensis, B. cytotoxicus, B. toyonensis*, and *B. weihenstephanensis*, are highly conserved. In particular, the N-terminal region from the fourth amino acid (Trp) to the ninth amino acid (Glu) is 100% conserved among the *B. cereus* group. In contrast, CdaS protein sequences in the *B. subtilis* group (*B. amyloliquefaciens, B. atrophaeus*, and *B. subtilis*) were more distant from each other.

However, the arrangement of *cdaS* gene sequences is very different among species in the genus *Bacillus* (Figure S15). In the *B. subtilis* group, *cdaS* genes are usually located on the negative-strand (−) DNA and upstream of a *yojK* gene which encodes glycosyltransferase. In contrast, *cdaS* genes in the *B. cereus* group are located on the positive-strand (+) DNA, and its adjacent genes encode phosphoglucomutase and leucyl aminopeptidase. The transcriptional regulation of *cdaS* genes in the *B. cereus* group and the *B. subtilis* group might be quite different due to the use of different promoter. Accordingly, *B. subtilis cdaS* is controlled by σ^G^ (Nicolas et al., 2012; Mehne et al., 2013), while the transcription of *B. thuringiensis cdaS* is under the control of σ^H^. This difference enables c-di-AMP generated by CdaS to function at different stages of sporulation likely through distinct receptors in *B. thuringiensis* and *B. subtilis*. More specifically, the c-di-AMP formed by *B. subtilis* CdaS is required for efficient germination (Mehne et al., 2014), whereas, c-di-AMP formed by *B. thuringiensis* CdaS is essential for sufficient sporulation.

### c-di-AMP regulation network in *B. thuringiensis*

**Figure 9:**
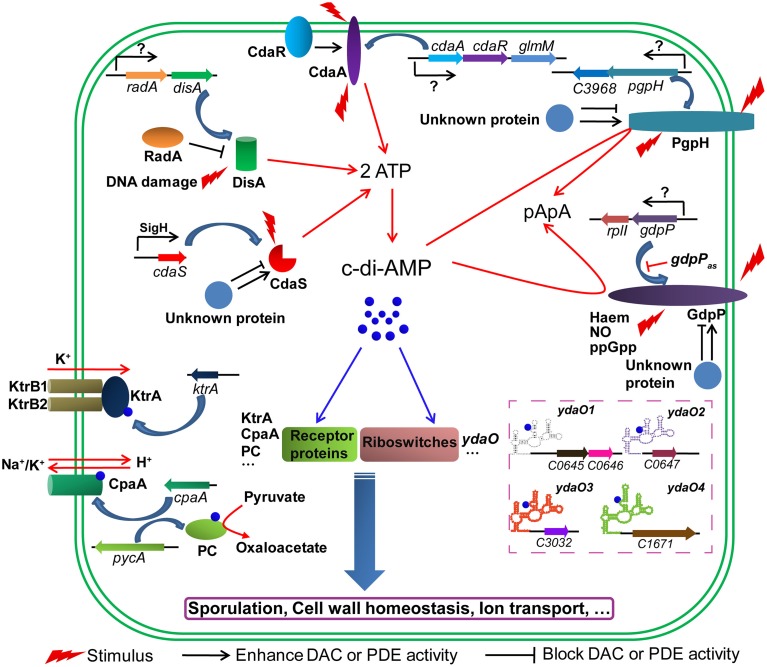
**Proposed signaling network mediated by c-di-AMP in *B. thuringiensis***. c-di-AMP is synthesized by DACs (DisA, CdaA and CdaS) and hydrolyzed by c-di-AMP-specific PDEs (GdpP and PgpH). Its level is spatially and temporally controlled by factors that affect these enzymes with respect to transcription, translation and enzymatic activity levels. c-di-AMP binds to receptor proteins and riboswitches and regulates various physiological processes. RadA, DNA repair protein; CdaR; CdaA regulator; GlmM, phosphoglucosamine mutase; RplI, ribosomal protein L9; *gdpP*_*as*_, *cis*-acting antisense RNA for *gdpP*; KtrA, potassium uptake protein; CapA, a predicted cation/proton antiporter; PC, pyruvate carboxylase; *ydaO*, c-di-AMP riboswitch, the structure of four predicted c-di-AMP riboswitches are shown; KtrB, a potassium uptake protein, interacts with KtrA to form a complex for potassium transport.

Both *B. thuringiensis* and *B. subtilis* possess three DACs and two c-di-AMP-specific PDEs. These enzymes are multi-domain proteins which indicate that they may respond to diverse signals to flexibly control the synthesis and degradation of c-di-AMP. The ability of DisA to synthesize c-di-AMP is regulated in at least two ways. DisA not only binds to DNA damage sites but also recruits other proteins such as RadA, which further reduces c-di-AMP synthesis (Witte et al., 2008; Zhang and He, 2013). The DAC activity of CdaA is governed by CdaR, whose encoding gene *cdaR* is located in the same operon with *cdaA* (Mehne et al., 2013). This operon is frequently extended to include the phosphoglucosamine mutase gene (*glmM*), which encodes a protein involved in one of the initial steps of peptidoglycan synthesis (Corrigan and Gründling, 2013; Gundlach et al., 2015b). Thus it is believed that c-di-AMP originated from CdaA could participate in cell wall synthesis and homeostasis (Luo and Helmann, 2012b). As a transmembrane protein, it also seems plausible that CdaA is able to sense a still undiscovered signal in order to directly regulate its DAC activity (Corrigan and Gründling, 2013). Similarly, the c-di-AMP-specific PDE GdpP is also a transmembrane protein containing a PAS domain and a highly modified GGDEF domain. Moreover, *gdpP* is found in an operon with *rplI*, the gene encoding ribosomal protein L9. It is possible that the PDE activity of GdpP is regulated through protein-protein interaction or sensing various stimuli (Rao et al., 2011; Corrigan and Gründling, 2013; Tan et al., 2013). In *B. subtilis*, the expression of *gdpP* is regulated by a cis-acting antisense (*gdpP*_*as*_) transcript which is under the control of σ^D^ (Luo and Helmann, 2012a). PgpH, a newly reported c-di-AMP-specific PDE (Huynh et al., 2015), consists of an N-terminal extracellular domain, seven transmembrane helices and a C-terminal catalytic HD domain (Figure S5A). The deletion of the *pgpH* improved the intracellular c-di-AMP level in *B. subtilis* (Gundlach et al., 2015b). The factors that affect PDE activity of PgpH and its physiological role are worthy to be confirmed and further investigated in the genus *Bacillus*. Compared with the above-mentioned enzymes, CdaS is likely specific to the order Bacillales, and its encoding gene *cdaS* is in a single-gene transcription unit. However, the DAC activity of CdaS could also be modulated by interactions with unknown proteins, and the N-terminal YojJ domain is also able to sense an unknown intracellular signal in order to regulate its DAC activity (Corrigan and Gründling, 2013). Hence, factors that spatially and temporally control the c-di-AMP level seem to be complex, but are closely connected and more efforts should be devoted to their study (Figure 9).

Among all the identified c-di-AMP receptor proteins, *B. thuringiensis* harbors homologs of KtrA, PC, and CpaA and the physiological functions mediated by these three c-di-AMP receptor proteins might also be conserved in *B. thuringiensis* (Figure 9). However, homologs of DarR, DarA and PstA are absent in *B. thuringiensis*, so it will be fruitful to identify new c-di-AMP receptor proteins to fully understand c-di-AMP-mediated signaling pathways, such as the downstream receptor and effector proteins that affect sporulation and the formation of parasporal crystals. Fortunately, some c-di-AMP targets in *B. thuringiensis* were obtained by affinity method (unpublished data). In addition to receptor proteins, the predicted *ydaO* riboswitch was found as a c-di-AMP receptor that seems to be conserved in bacteria. In BMB171, four *ydaO* riboswitches were annotated in the Rfam database (Figure 9), and further study on the functions of these four c-di-AMP riboswitches may enrich the c-di-AMP regulation pathway.

### Conflict of interest statement

The authors declare that the research was conducted in the absence of any commercial or financial relationships that could be construed as a potential conflict of interest.
