# Supplementary material for: Functional analysis of the sporulation-specific diadenylate cyclase CdaS in Bacillus thuringiensis
Source: Front Microbiol. 2015 Sep 14;6:908. doi: 10.3389/fmicb.2015.00908 (PMC4568413; doi:10.3389/fmicb.2015.00908)
Supplement: Supplementary file 1 [file Table1.DOC]

**TABLE S1. Primers used in this study**

| **Primers** | **Primer sequences (5'-3')** | **Restriction sites** |
| --- | --- | --- |
| *cdaS*-F | CATGCCATGGGACACGAATGGGGCTTG | NcoI |
| *cdaS*-R | TCGACTCGAGTAAAGAAATCGTATAC | XhoI |
| *cdaS*_70-201_-F | CATGCCATGGGAACGGAAAGTTCATCT | NcoI |
| *cdaS*_28-201_-F | TGCACCATGGGAAGGAACGCAATCGATA | NcoI |
| *cdaS*_17-201_-F | TGCACCATGGGAATTGAAATTGCTGAA | NcoI |
| *cdaS*_11-201_-F | TGCACCATGGGAAAAATACAAACAAAG | NcoI |
| *cdaS*_10-201_-F | TGCACCATGGGACTCAAAATACAAACA | NcoI |
| *cdaS*_9-201_-F | TGCACCATGGGAGAGCTCAAAATACAA | NcoI |
| *cdaS*_8-201_-F | TGCACCATGGGAGAAGAGCTCAAAATA | NcoI |
| *cdaS*_7-201_-F | TGCACCATGGGATCAGAAGAGCTCAAA | NcoI |
| *cdaS*_5-201_-F | TGCACCATGGGATTGTCAGAAGAGCTC | NcoI |
| *cdaS*_4-201_-F | TGCACCATGGGATGGGGCTTGTCAGAA | NcoI |
| *cdaS*_3-201_-F | TGCACCATGGAATGGGGCTTGTCA | NcoI |
| *cdaS*_W4G_-F | CATGCCATGGGACACGAAGGAGGCTTG | NcoI |
| *cdaS*_W4A_-F | CATGCCATGGGACACGAAGCTGGCTTG | NcoI |
| *cdaS*_W4D_-F | CATGCCATGGGACACGAAGATGGCTTG | NcoI |
| *cdaS*_W4K_-F | CATGCCATGGGACACGAAAAGGGCTTG | NcoI |
| *cdaS*_DGA_-F | AACCCTCTTCATGCCGCCGCCGTTCTCGT |  |
| *cdaS*_DGA_-R | ACGAGAACGGCGGCGGCATGAAGAGGGTT |  |
| *cdaS*_RHR_-F | AGGAACAGCCGCCGCCGCTGCAATTGGCTT |  |
| *cdaS*_RHR_-R | AAGCCAATTGCAGCGGCGGCGGCTGTTCCT |  |
| *cdaA*_86-273_-F | CATGCCATGGCGAAGAGCACTTGAGCAGC | NcoI |
| *cdaA*_86-273_-R | TCGACTCGAGTCCATGACGCTTTCTCCTC | XhoI |
| *P_cdaS_*-*lacZ*-F | CCATGGAAACACATATGTTGTGATACC | NcoI |
| *P_cdaS_*-*lacZ*-R | AGATCTAATCCTCATTCCCTTTAGTGGA | BglII |
| *P_disA_*-*lacZ*-F | CCATGGATGGTGCTCGTCCACTACGTAGAG | NcoI |
| *P_disA_*-*lacZ*-R | GGATCCATCTTTTTATATTTCACTTCGCTTT | BamHI |
| *P_cdaA_*-*lacZ*-F | CCATGGAAATGTAAGAAAAGCAACTGAGA | NcoI |
| *P_cdaA_*-*lacZ*-R | GGATCCGCTATCCCTTCCTCCTACTTACGAG | BamHI |
| *P_gdpP_*-*lacZ*-F | CCATGGACTGCAAGGTCTGACATTTATCGC | NcoI |
| *P_gdpP_*-*lacZ*-R | GGATCCGTATACAACTCCTATAAAGTAAAC | BamHI |
| *cdaS*-U-F | CTGAACGCGTGTCACGAATGGCATACTC | MluI |
| *cdaS*-U-R | CGACGGATCCAATCCTCATTCCCTTTAG | BamHI |
| *cdaS*-D-F | GGATCCAGTGAAACTTTAATCAGCCCTC | BamHI |
| *cdaS*-D-R | GGTACCGACAAAGCTTGCTGAGTATGCTG | KpnI |
| CU-F | GGATCCAAACACATATGTTGTGATACCG | BamHI |
| CD-R | GAATTCAACACTTGTGGAGCGTTTTGGAG | EcoRI |
| C-F | CATGCCATGGGACACGAATGGGGCTTG | NcoI |
| C-R | TCGACTCGAGTAAAGAAATCGTATAC | XhoI |
| *cdaA*-U-F | CCCAAGCTTTTCACCATCTGGAAATATTCAT | HindIII |
| *cdaA*-U-R | CGACGCGTGCTATCCCTTCCTCCTACTTAC | MluI |
| *cdaA*-D-F | CGACGCGTATGGATAAGTTAATGGAGAATC | MluI |
| *cdaA*-D-R | CGGGATCCGCTAAACTCCCCTTCTTCGTATA | BamHI |
| *cdaA*-F | CGCGGATCCATGCCTTTTGAAGATACGACCA | BamHI |
| *cdaA*-R | CCGCTCGAGTTATCCATGACGCTTTCTCCTC | XhoI |
| *disA*-U-F | AAGCTTTCCCAATTTTCATCGTAGGACATGT | Hind III |
| *disA*-U-R | GGATCCAGCCTAGCCTCCTAATGTCAAACG | BamH I |
| *disA*-D-F | GGATCCGAATATTACATTGATTCTATAATAT | BamH I |
| *disA*-D-R | GGTACCACAATTGTTCCTTCTAAAAACTTTG | Kpn I |
| *disA*-F | CATGCCATGGAAGAAAATAAGCAACGTGTC | NcoI |
| *disA*-R | CTAGCTCGAGATTCTCTCTACTCATATAGAG | XhoI |
| *sigH*-U-F | AAGCTTAGTAATTGGTAGTGAAGGAA | HindIII |
| *sigH*-U-R | GGATCCCTTGATCCCTCCGACCGCTAT | BamHI |
| *sigH*-D-F | GGATCCGAAGTAACAAGTGCTACAG | BamHI |
| *sigH*-D-R | GGTACCCCGGGACAACAACACGGAA | KpnI |
| *sigH*-F | GTGGAAGCAGGCTTCGTAAGTATAG |  |
| *sigH*-R | CTCTCATTTCCATATATCGTTCCAA |  |
| *sigF*-U-F | AAGCTTATCTAGTCCTTGCATAGCACCTTCC | HindIII |
| *sigF*-U-R | GGATCCTTGTCTATTCGATTTTTTTATGCCG | BamHI |
| *sigF*-D-F | GGATCCAGGCTATTCTCCTTAATTGCATAGA | BamHI |
| *sigF*-D-R | GGTACCGAGGGTGAATTAGATCATCATACGG | KpnI |
| *sigF*-F | ATGGACATAGAGGTCAAAAATGAGA |  |
| *sigF*-R | TTATTCGTCTATTCGATCTTTCATC |  |
| *sigE*-U-F | ACGCGTCCCACTGCTCGTCCTTTTGTTTTTC | MluI |
| *sigE*-U-R | GTCGACAGAATAAGACCAGCCGTTATGTAGG | SalI |
| *sigE*-D-F | GTCGACATTATCCCGCCTCTCATTTATTAAG | SalI |
| *sigE*-D-R | GGTACCGAAACTACTGTACTCGTTACTTATT | KpnI |
| *sigE*-F | ATGATGAAATTAAAATTTTATTT |  |
| *sigE*-R | TTACACCATTTTATTAAATTCTTT |  |
| 1028-R | GTGCGAATAAGGGACAGTGAAGAAGG |  |
