# Supplementary material for: Functional analysis of the sporulation-specific diadenylate cyclase CdaS in Bacillus thuringiensis
Source: Front Microbiol. 2015 Sep 14;6:908. doi: 10.3389/fmicb.2015.00908 (PMC4568413; doi:10.3389/fmicb.2015.00908)
Supplement: Supplementary file 2 [file Table2.DOC]

**TABLE S2.** **The distribution of c-di-AMP related genes in genus *Bacillus***

| **Subgenus** | **Species** | **Strains** | ***disA*** | ***cdaA*** | ***cdaS*** | ***gdpP*** | ***pgpH*** |
| --- | --- | --- | --- | --- | --- | --- | --- |
|  | *B. cellulosilyticus* | *B. cellulosilyticus* DSM 2522 | + | + | - | + | + |
| *B. cereus* group | *B. anthracis* | *B. anthracis* str. 'Ames Ancestor' | + | + | + | + | + |
|  |  | *B. anthracis* str. A0248 | + | + | + | + | + |
|  |  | *B. anthracis* str. Ames | + | + | + | + | + |
|  |  | *B. anthracis* str. CDC 684 | + | + | + | + | + |
|  |  | *B. anthracis* str. H9401 | + | + | + | + | + |
|  |  | *B. anthracis* str. Sterne | + | + | + | + | + |
|  | *B. cereus* | *B. cereus* 03BB102 | + | + | + | + | + |
|  |  | *B. cereus* AH187 | + | + | + | + | + |
|  |  | *B. cereus* AH820 | + | + | + | + | + |
|  |  | *B. cereus* ATCC 10987 | + | + | + | + | + |
|  |  | *B. cereus* ATCC 14579 | + | + | + | + | + |
|  |  | *B. cereus* B4264 | + | + | + | + | + |
|  |  | *B. cereus* biovar anthracis str. CI | + | + | + | + | + |
|  |  | *B. cereus* E33L | + | + | + | + | + |
|  |  | *B. cereus* F837/76 | + | + | + | + | + |
|  |  | *B. cereus* FRI-35 | + | + | + | + | + |
|  |  | *B. cereus* G9842 | + | + | + | + | + |
|  |  | *B. cereus* NC7401 | + | + | + | + | + |
|  |  | *B. cereus* Q1 | + | + | + | + | + |
|  | *B. cytotoxicus* | *B. cytotoxicus* NVH 391-98 | + | + | + | + | + |
|  | *B. thuringiensis* | *B. thuringiensis* str. Al Hakam | + | + | + | + | + |
|  |  | *B. thuringiensis* BMB171 | + | + | + | + | + |
|  |  | *B. thuringiensis* Bt407 | + | + | + | + | + |
|  |  | *B. thuringiensis* HD-771 | + | + | + | + | + |
|  |  | *B. thuringiensis* HD-789 | + | + | + | + | + |
|  |  | *B. thuringiensis* MC28 | + | + | + | + | + |
|  |  | *B. thuringiensis* serovar chinensis CT-43 | + | + | + | + | + |
|  |  | *B. thuringiensis* serovar finitimus YBT-020 | + | + | + | + | + |
|  |  | *B. thuringiensis* serovar thuringiensis str. IS5056 | + | + | + | + | + |
|  |  | *B. thuringiensis* serovar konkukian str. 97-27 | + | + | + | + | + |
|  |  | *B. thuringiensis* serovar kurstaki str. HD73 | + | + | + | + | + |
|  |  | *B. thuringiensis* YBT-1518 | + | + | + | + | + |
|  | *B. toyonensis* | *B. toyonensis* BCT-7112 | + | + | + | + | + |
|  | *B. weihenstephanensis* | *B. weihenstephanensis* KBAB4 | + | + | + | + | + |
|  | *B. clausii* | *B. clausii* KSM-K16 | + | + | - | + | + |
|  | *B. coagulans* | *B. coagulans* 2-6 | - | + | - | + | + |
|  |  | *B. coagulans* 36D1 | - | + | - | + | + |
|  | *B. halodurans* | *B. halodurans* C-125 | + | + | - | + | + |
|  | *B. infantis* | *B. infantis* NRRL B-14911 | + | + | + | + | + |
|  | *B. megaterium* | *B. megaterium* DSM 319 | + | + | + | + | + |
|  |  | *B.* *megaterium* QM B1551 | + | + | + | + | + |
|  |  | *B. megaterium* WSH-002 | + | + | + | + | + |
|  | *B. pseudofirmus* | *B. pseudofirmus* OF4 | + | + | - | + | + |
|  | *B. pumilus* | *B. pumilus* SAFR-032 | + | + | + | + | + |
| *B. subtilis* group | *B. amyloliquefaciens* | *B. amyloliquefaciens* CC178 | + | + | + | + | + |
|  |  | *B. amyloliquefaciens* DSM 7 | + | + | + | + | + |
|  |  | *B. amyloliquefaciens* subsp. plantarum str. FZB42 | + | + | + | + | + |
|  |  | *B. amyloliquefaciens* IT-45 | + | + | + | + | + |
|  |  | *B. amyloliquefaciens* LFB112 | + | + | + | + | + |
|  |  | *B. amyloliquefaciens* LL3 | + | + | + | + | + |
|  |  | *B. amyloliquefaciens* subsp. plantarum AS43.3 | + | + | + | + | + |
|  |  | *B. amyloliquefaciens* subsp. plantarum CAU B946 | + | + | + | + | + |
|  |  | *B. amyloliquefaciens* subsp. plantarum NAU-B3 | + | + | + | + | + |
|  |  | *B. amyloliquefaciens* subsp. plantarum UCMB5033 | + | + | + | + | + |
|  |  | *B. amyloliquefaciens* subsp. plantarum UCMB5036 | + | + | + | + | + |
|  |  | *B. amyloliquefaciens* subsp. plantarum UCMB5113 | + | + | + | + | + |
|  |  | *B. amyloliquefaciens* subsp. plantarum YAU B9601-Y2 | + | + | + | + | + |
|  |  | *B. amyloliquefaciens* TA208 | + | + | + | + | + |
|  |  | *B. amyloliquefaciens* XH7 | + | + | + | + | + |
|  |  | *B. amyloliquefaciens* Y2 | + | + | + | + | + |
|  | *B. atrophaeus* | *B. atrophaeus* 1942 | + | + | + | + | + |
|  | *B. licheniformis* | *B. licheniformis* 9945A | + | + | - | + | + |
|  |  | *B. licheniformis* DSM 13 = ATCC 14580 | + | + | - | + | + |
|  | *B. subtilis* | *B. subtilis* subsp. subtilis str. 168 | + | + | + | + | + |
|  |  | *B. subtilis* subsp. subtilis 6051-HGW | + | + | + | + | + |
|  |  | *B. subtilis* subsp. subtilis str. BAB-1 | + | + | + | + | + |
|  |  | *B. subtilis* BSn5 | + | + | + | + | + |
|  |  | *B. subtilis* subsp. subtilis str. BSP1 | + | + | + | + | + |
|  |  | *B. subtilis* subsp. natto BEST195 | + | + | + | + | + |
|  |  | *B. subtilis* PY79 | + | + | + | + | + |
|  |  | *B. subtilis* QB928 | + | + | + | + | + |
|  |  | *B. subtilis* subsp. subtilis str. RO-NN-1 | + | + | + | + | + |
|  |  | *B. subtilis* subsp. spizizenii TU-B-10 | + | + | + | + | + |
|  |  | *B. subtilis* subsp. spizizenii str. W23 | + | + | + | + | + |
|  |  | *B. subtilis* XF-1 | + | + | - | + | + |
|  |  | *B.* sp. 1NLA3E | - | + | - | + | + |
|  |  | *B.* sp. JS | + | + | + | + | + |

+: presence; -: absence
