# Supplementary material for: Functional analysis of the sporulation-specific diadenylate cyclase CdaS in Bacillus thuringiensis
Source: Front Microbiol. 2015 Sep 14;6:908. doi: 10.3389/fmicb.2015.00908 (PMC4568413; doi:10.3389/fmicb.2015.00908)
Supplement: Supplementary file 3 [file Image1.PDF]

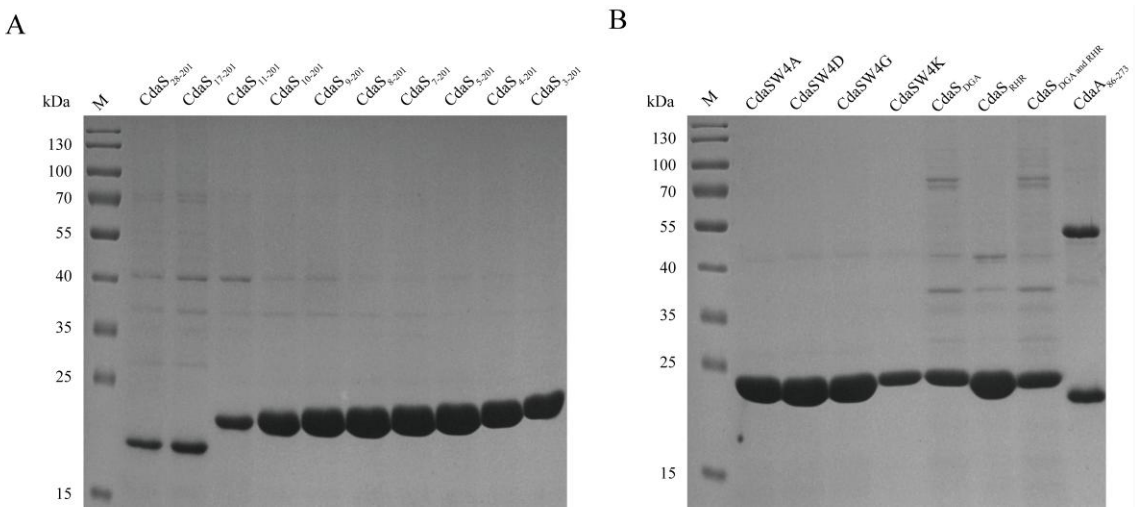

Figure S1. The SDS-PAGE images of related proteins used in this study. Proteins were separated by 12% SDS-PAGE, Lane M is the PageRulerTM Prestained Protein Ladder Marker (Thermo Scientific).
