# Supplementary material for: Functional analysis of the sporulation-specific diadenylate cyclase CdaS in Bacillus thuringiensis
Source: Front Microbiol. 2015 Sep 14;6:908. doi: 10.3389/fmicb.2015.00908 (PMC4568413; doi:10.3389/fmicb.2015.00908)
Supplement: Supplementary file 4 [file Image2.PDF]

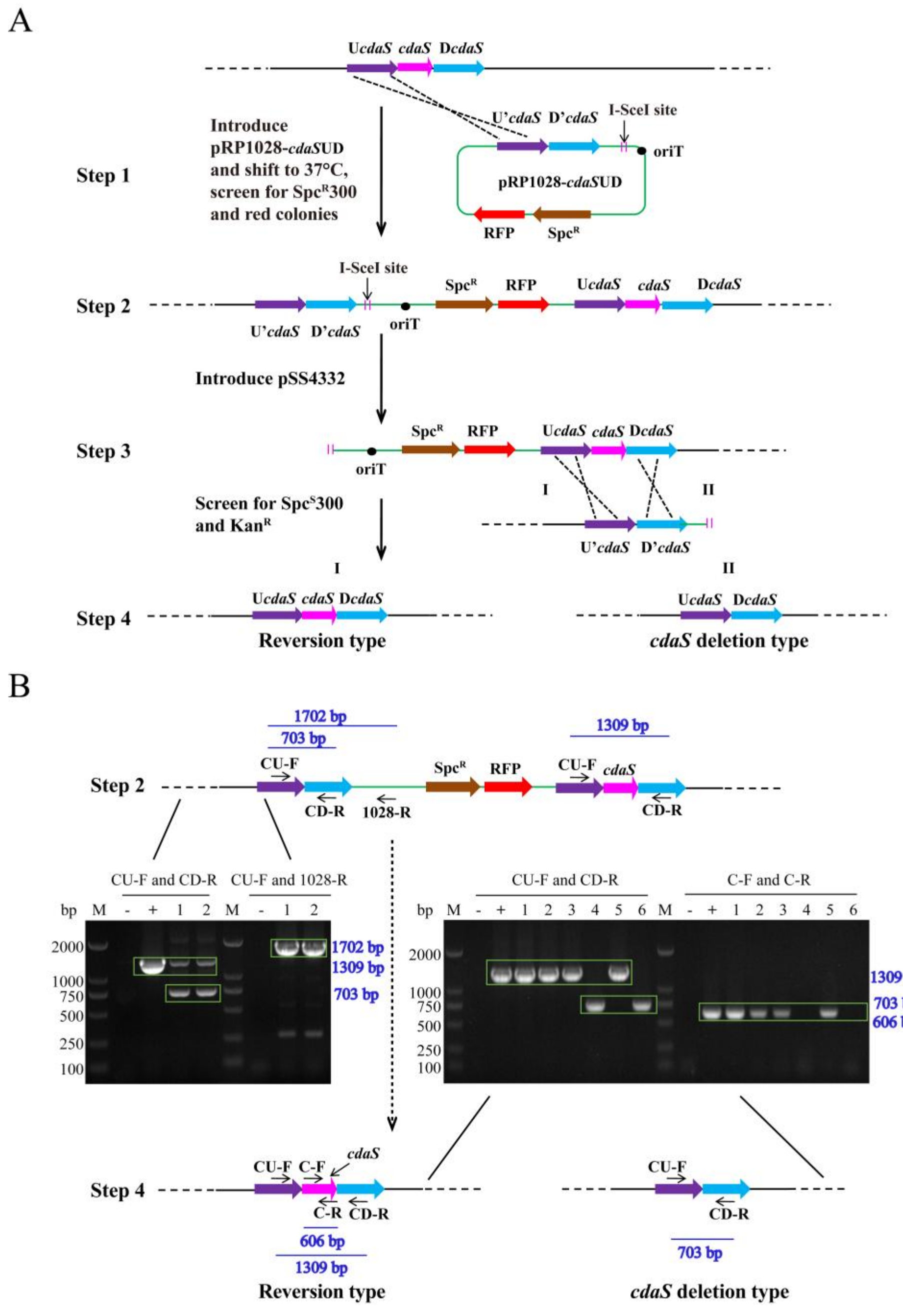

Figure S2. (A) Schematic diagram of I-SceI-dependent gene knock-out procedures in *B. thuringiensis* BMB171. For Step 1, *B. thuringiensis* BMB171 (recipient strain), *E. coli* DH5α containing pRP1028-*cdaS*UD (donor strain) and *E. coli* DH5α containing helper plasmid pSS1827 (helper strain) were grown in LB medium overnight, and then 1.5 mL of the three cultures were collected and washed three times with 1 mL LB medium, and resuspended in 0.2 mL LB medium, respectively. Afterwards, 50 μL of each of three cultures was mixed together, and then poured on a LB plate. After 24 h culture at 28°C , the regenerated strain colonies were scraped and resuspended in 500 μL LB medium, and then spread on LB plates containing spectinomycin (300 μg/mL) and polymyxin (60 units), and finally cultured at 28°C for about 6-7

days. During this period, the integrating plasmid pRP1028-*cdaS*UD was integrated into BMB171 genome by homologous recombination, rendering single cross-over strains turned into red color. The redundant pRP1028-*cdaS*UD plasmid in the single cross-over strain was eliminated by shifting incubation temperature to 37°C through two continuous passages and for each passage, shake time was 6 h. In Step 2, the acquired red color single cross-over strains were confirmed by PCR using primers (**Table S1**). In Step 3, the expression plasmid pSS4332 (in DH5α-pSS4332), which produced the restriction endonuclease I-SceI, was conjugative transferred into the single cross-over insertion strain by triparental mating with the help of *E. coli* DH5α-pSS1827 as described in Step 1except that the screening condition was on LB plates containing kanamycin (50 μg/mL) and polymyxin (60 units) and the incubation time was only one day. Under the circumstances, expression of I-SceI by pSS4332 resulted in the cleavage of the integrated genome at the I-SceI recognition site, producing a double strand break in the chromosome. This DNA break stimulated the host recombination/repair machinery to repair it. Ultimately, the strains that undergone the second recombination lost the spectinomycin (300 μg/mL) resistance. So in Step 4, colonies with spectinomycin (300 μg/mL) sensitivity and kanamycin resistance were selected in which there were two possibilities: generating gene deletion mutant or restoring to parent strain. The Δ*cdaS* was verified by PCR amplification (**Figure S2B**). Finally, the redundant expression plasmid pSS4332 in Δ*cdaS* was removed using continuous passage at 28°C U*cdaS* and D*cdaS* represent the upstream and downstream sequences of *cdaS* gene, respectively, whereas U'*cdaS* and D'*cdaS* are the homologous fragments of U*cdaS* and D*cdaS*, respectively, which were located in integrating plasmid pRP1028-*cdaS*UD; Spc^R300: spectinomycin resistance at the concentration of 300 μg/mL; Spc^S300: spectinomycin sensitivity at the concentration of 300 μg/mL; Kan^R: kanamycin resistance at the concentration of 50 μg/mL. **(B) PCR verification that needed in steps 2 and 4.** The sequences of primers that indicated in this image are list in **Table S1**. The DNA image on the left was PCR verification result in step 2 in which the pRP1028-*cdaS*UD was integrated into the chromosome of BMB171 through the first homologous single exchange at the upstream region of *cdaS*; the right DNA image is the PCR result for screening *cdaS* mutant strain after the second homologous single exchange.
