# Supplementary material for: Functional analysis of the sporulation-specific diadenylate cyclase CdaS in Bacillus thuringiensis
Source: Front Microbiol. 2015 Sep 14;6:908. doi: 10.3389/fmicb.2015.00908 (PMC4568413; doi:10.3389/fmicb.2015.00908)
Supplement: Supplementary file 5 [file Image3.PDF]

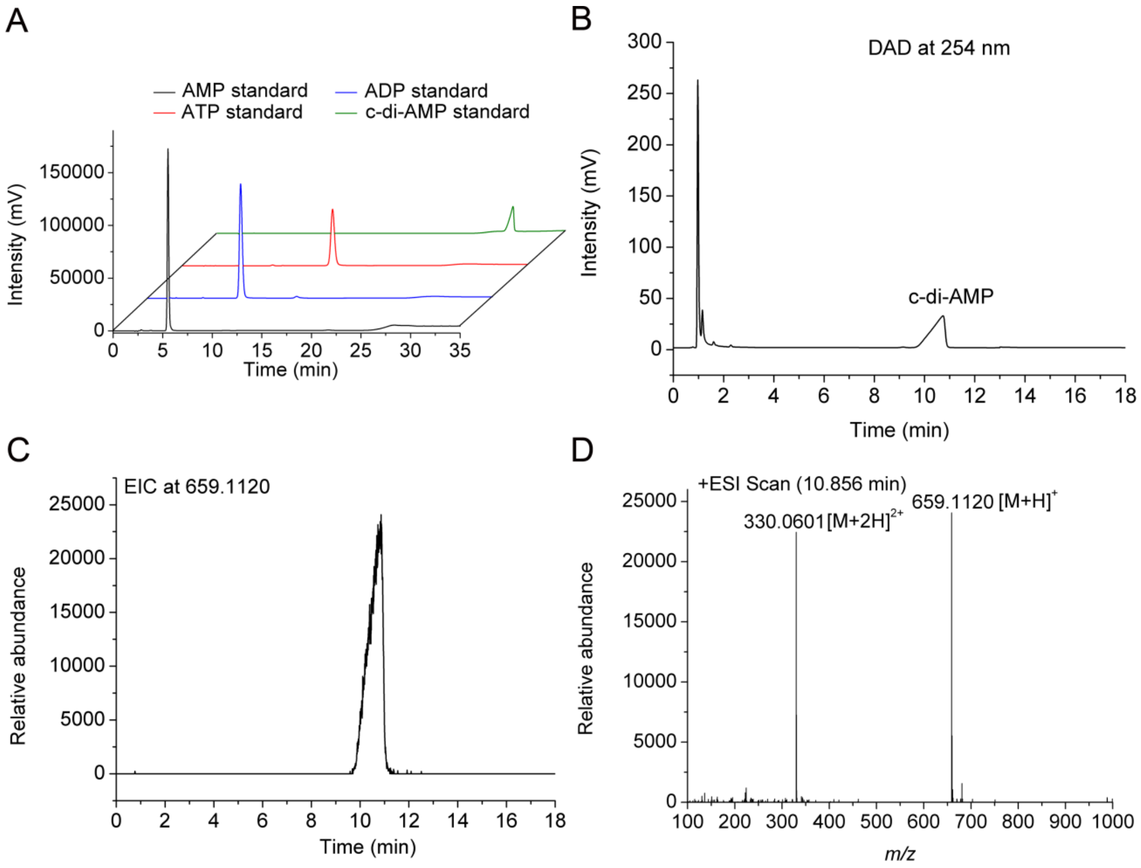

Figure S3. (A) HPLC Chromatograms of various nucleoside standards. Black: 100 µM AMP; blue: 200 µM ADP; red: 200 µM ATP; green: 100 µM c-di-AMP. **(B-D) Identification of c-di-AMP by LC/Q-TOF.** (B) The HPLC chromatogram of DAC reaction of CdaS. 1 µM CdaS was incubated with standard reaction mixture at 37 ^{o}C for 10 min in 100 µL reaction system; the wavelength of the diode array detector (DAD) was set at 254 nm. (C) The corresponding extracted ion chromatorgraphy (EIC) at m/z = 659.1120 for c-di-AMP. (D) The mass spectrum of c-di-AMP that related to B. *m/z* = 659.1120 and 330.0601 are the singly ($[M+H]^{+}$) and doubly protonated ($[M+2H]^{2+}$) species of c-di-AMP.
