# Supplementary material for: Functional analysis of the sporulation-specific diadenylate cyclase CdaS in Bacillus thuringiensis
Source: Front Microbiol. 2015 Sep 14;6:908. doi: 10.3389/fmicb.2015.00908 (PMC4568413; doi:10.3389/fmicb.2015.00908)
Supplement: Supplementary file 6 [file Image4.PDF]

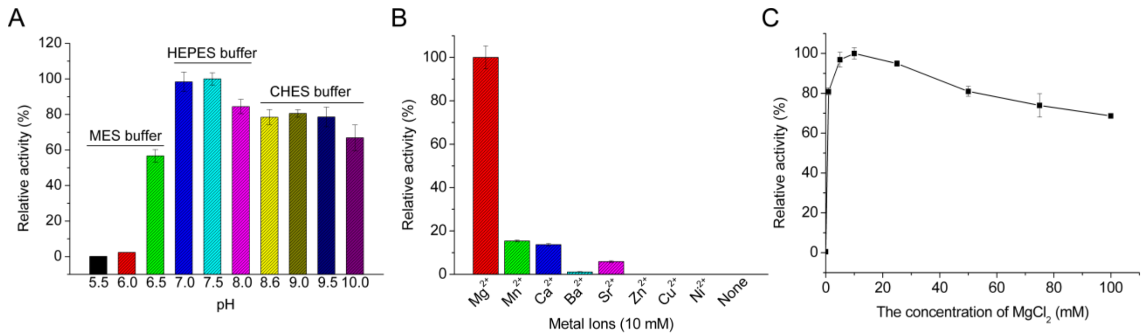

Figure S4. Enzymatic properties of CdaS. (A) Effect of pH on DAC activity of CdaS. 1 μM CdaS was incubated with standard reaction mixture (except buffer; MES: pH 5.5-6.5; HEPES: pH 7.0-8.0; and CHES: pH 8.6-10.0) at 37 °C for 2 h in 100 μL reaction system. The DAC activity at pH 7.5 was taken as 100%. (B) Effect of divalent metal ions on DAC activity of CdaS. 1 μM CdaS was incubated with standard reaction mixture (except for divalent metal ions) at 37 °C for 2 h in 100 μL reaction system. The DAC activity at 10 mM Mg^{2+} was taken as 100%. (C) Effect of Mg^{2+} concentration on DAC activity of CdaS. 1 μM CdaS was incubated with standard reaction mixture (except for the Mg^{2+} concentration) at 37 °C for 2 h in 100 μL reaction system. The DAC activity with 10 mM Mg^{2+} was taken as 100%.
