# Supplementary material for: Functional analysis of the sporulation-specific diadenylate cyclase CdaS in Bacillus thuringiensis
Source: Front Microbiol. 2015 Sep 14;6:908. doi: 10.3389/fmicb.2015.00908 (PMC4568413; doi:10.3389/fmicb.2015.00908)
Supplement: Supplementary file 7 [file Image5.PDF]

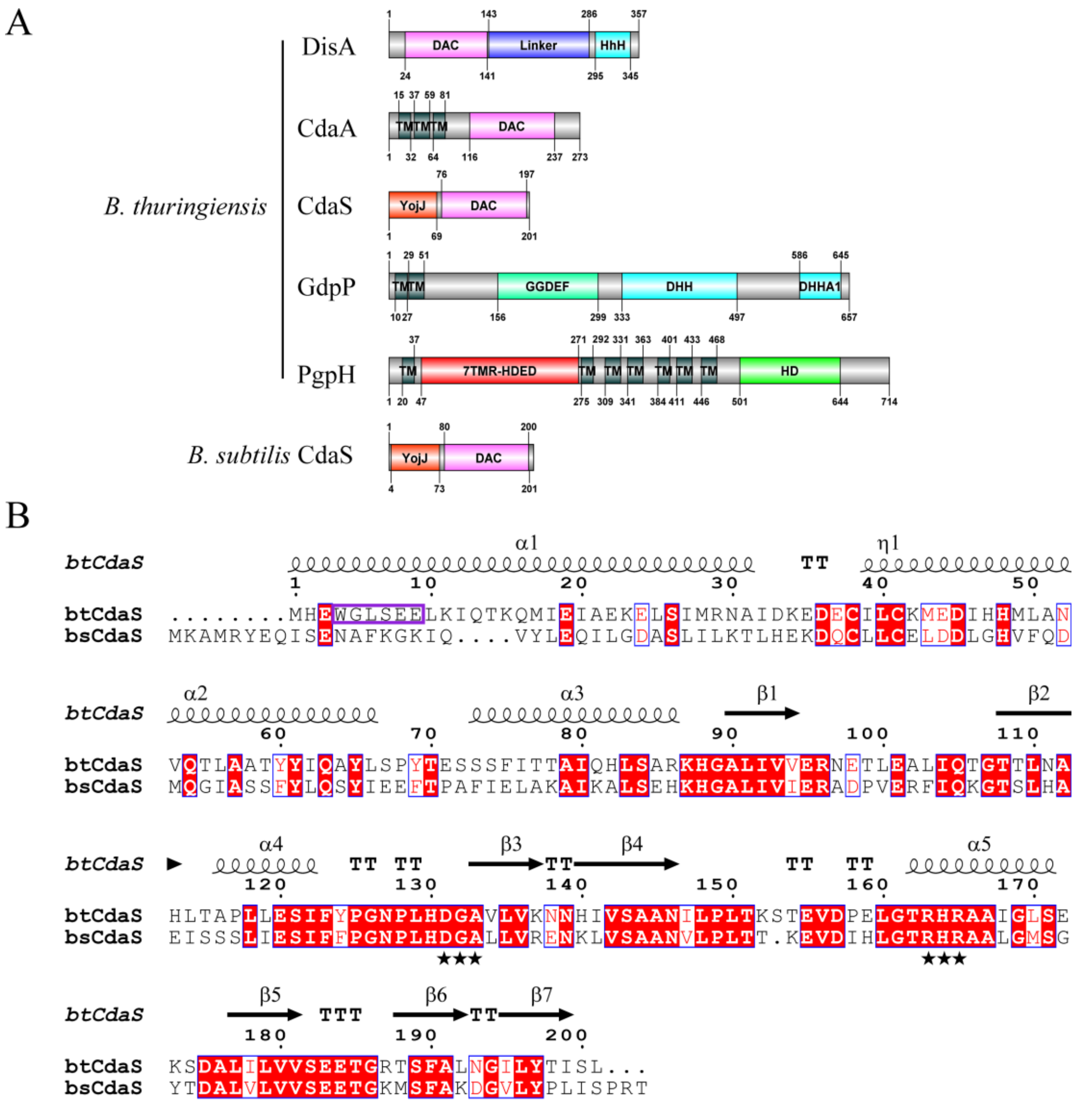

Figure S5. (A) Domain architecture of c-di-AMP related enzymes in *B. thuringiensis* and CdaS in *B. subtilis*. The domains of the six proteins were predicted in Pfam database, and the domains were drawn using DOG 1.0 (Ren et al., 2009). **(B) Full-length sequence alignment of *B. thuringiensis* CdaS (btCdaS) and *B. subtilis* CdaS (bsCdaS).** The sequence alignment was performed using ClustalW2, and the figure was made using ESPript (Gouet et al., 1999). The two conserved motifs for DAC activity are labeled with asterisks; meanwhile, the amino acids critical for the DAC activity of *B. thuringiensis* CdaS that found in this study are highlighted by purple box. The secondary structures of *B. thuringiensis* CdaS are shown at the top of the panel.

References

Ren, J., Wen, L., Gao, X., Jin, C., Xue, Y., and Yao, X. (2009). DOG 1.0: illustrator of protein domain structures. *Cell Res*. 19, 271-273.

Gouet, P., Courcelle, E., Stuart, D. I., and Métoz, F. (1999) ESPript: analysis of multiple sequence alignments in PostScript. *Bioinformatics* 15, 305-308.
