# Supplementary material for: Functional analysis of the sporulation-specific diadenylate cyclase CdaS in Bacillus thuringiensis
Source: Front Microbiol. 2015 Sep 14;6:908. doi: 10.3389/fmicb.2015.00908 (PMC4568413; doi:10.3389/fmicb.2015.00908)
Supplement: Supplementary file 8 [file Image6.PDF]

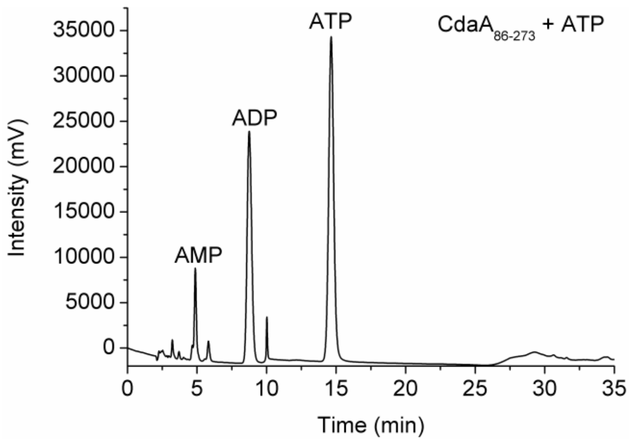

Figure S6. Determination the DAC activity of CdaA$_{86\text{-}273}$. 1 μM CdaA$_{86\text{-}273}$ was incubated with 100 mM Tris-HCl buffer (pH 8.0) containing 200 μM ATP and 10 mM $MgCl_2$ at 37 ^{o}C in 100 μL reaction system for overnight.
