# Supplementary material for: Functional analysis of the sporulation-specific diadenylate cyclase CdaS in Bacillus thuringiensis
Source: Front Microbiol. 2015 Sep 14;6:908. doi: 10.3389/fmicb.2015.00908 (PMC4568413; doi:10.3389/fmicb.2015.00908)
Supplement: Supplementary file 9 [file Image7.PDF]

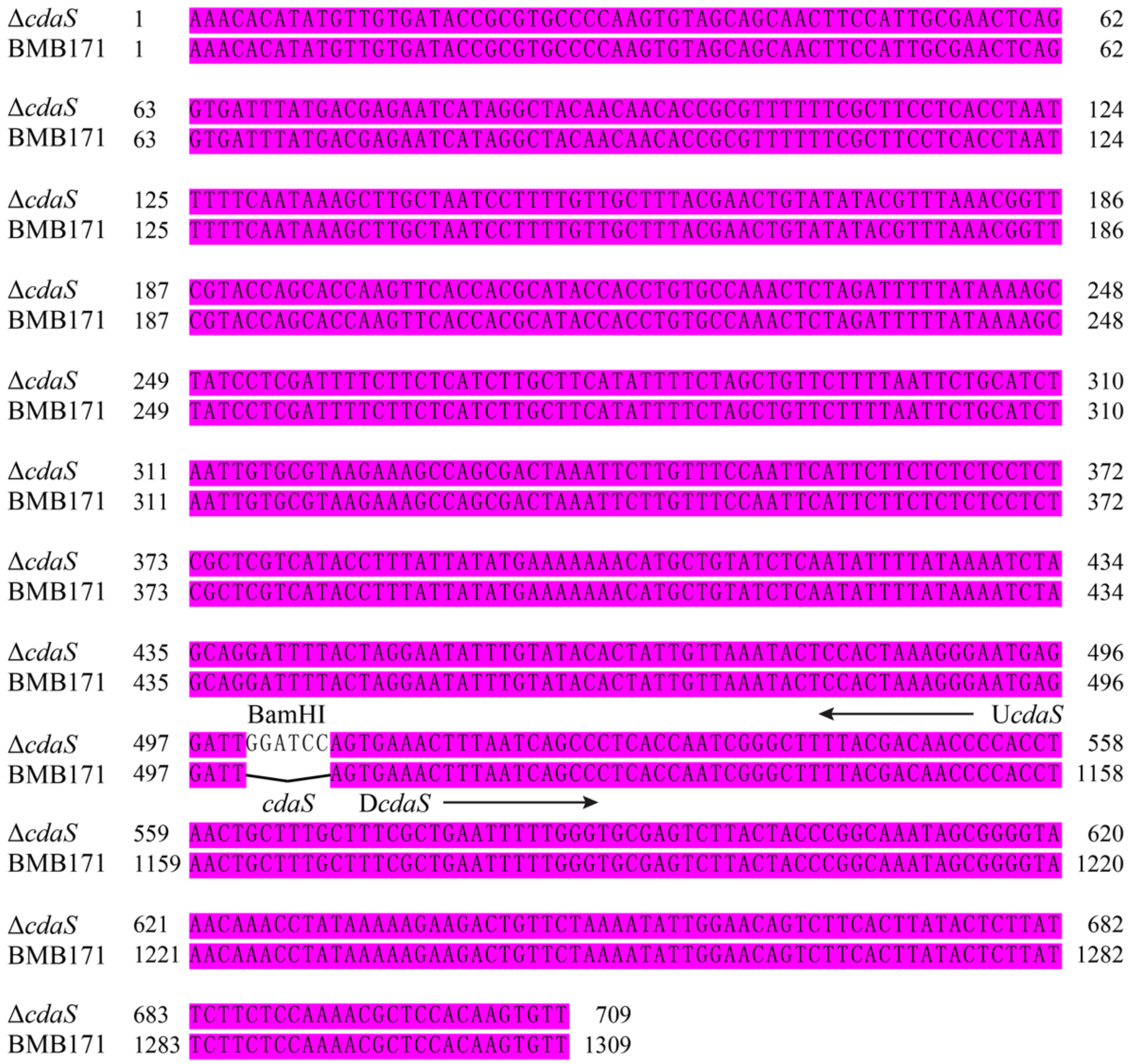

Figure S7. Verification of Δ*cdaS* by sequencing. Sequence alignment of PCR products amplified from the Δ*cdaS* genomic DNA and the BMB171 genomic DNA using primer pair *UcdaS* F/*DcdaS* R. The PCR products (about upstream 500 bp and downstream 200 bp sequences of *cdaS*) were shown. The restriction site of BamHI GGATCC residues in the *cdaS* locus of the BMB171 chromosome (NC_014171, GI: 296500838). What is missing is the *cdaS* gene complete sequence (*BMB171_C4535*, PID: 296505363, in the region 4746955..4747560 of NC_014171), and it is also listed as follows:

ATGcacgaatggggcttgtcagaagagctcaaaatacaaacaaagcaaatgattgaaattgctgaaaaagaactatcgattatgaggaacgcaatcgataaagaagacga
atgtattttatgcaaaatggaagatattcatcatatgttagcaaatgtacaaacattggcagctacatactatattcaggcatatttatcaccttatacggaaagttcatcttttattacga
cagctatccaacacttaagcgccagaaaacatggtgctcttatcgttgtggaaagaaacgagacgcttgaagctctcattcaaactggaacgacattaaacgctcatttaactgc
accattactcgaatcaatattttatccaggtaaccctcttcatgacggtgccgttctcgtaaaaaataatcatattgtctcagctgctaatattcttcctttaacgaaaagtacagaagtt
gatcctgagctaggaacacgtcacagagctgcaattggcttatcagaaaagagtgatgcacttatattagttgtctctgaagaaacgggccgtacttcttttgctttaaacgggatt
ttgtatacgatttctttta**TAA**
