# Supplementary material for: Functional analysis of the sporulation-specific diadenylate cyclase CdaS in Bacillus thuringiensis
Source: Front Microbiol. 2015 Sep 14;6:908. doi: 10.3389/fmicb.2015.00908 (PMC4568413; doi:10.3389/fmicb.2015.00908)
Supplement: Supplementary file 10 [file Image8.PDF]

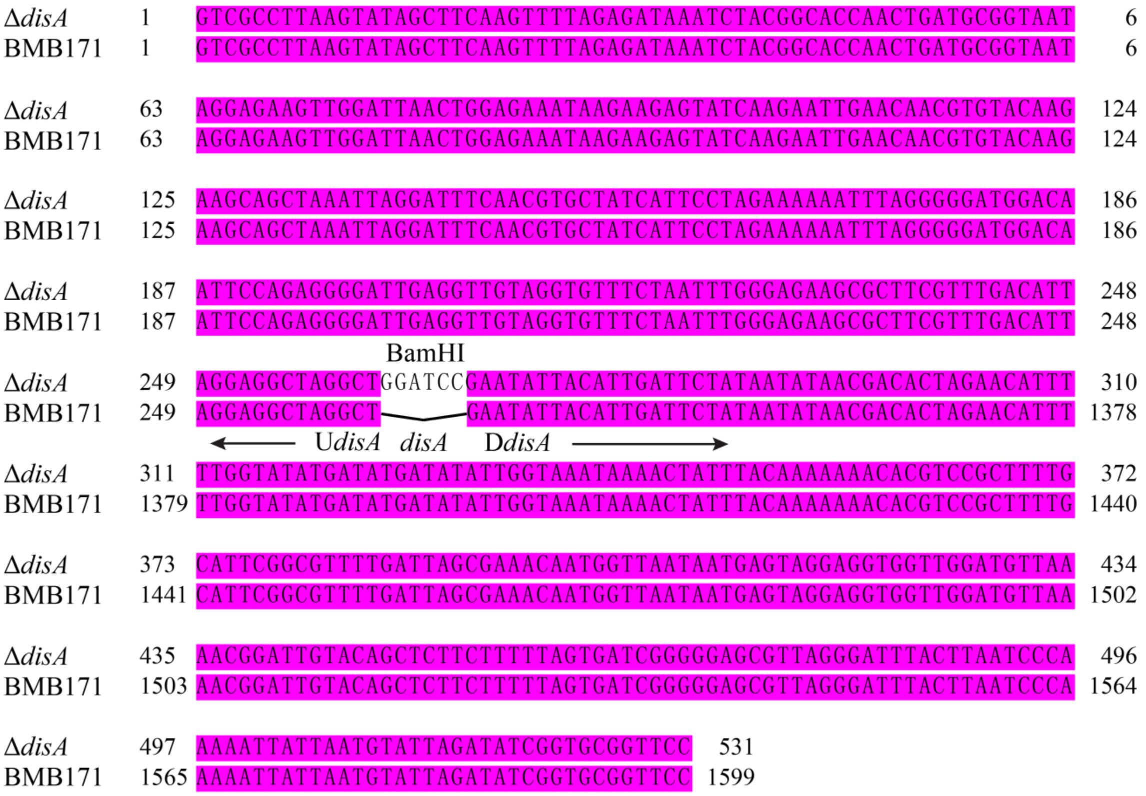

Figure S8. Verification of Δ*disA* by sequencing. Sequence alignment of PCR products amplified from the Δ*disA* genomic DNA and the BMB171 genomic DNA using primer pair *UdisA* F/*DdisA* R. The PCR products (about upstream 260 bp and downstream 260 bp sequences of *disA*) were shown. The restriction site of BamHI GGATCC residues in the *disA* locus of the BMB171 chromosome (NC_014171, GI: 296500838). What is missing is the *disA* gene complete sequence (*BMB171_C0080*, PID: 296500918, in the region 100457..101530 of NC_014171), and it is also listed as follows:

ATGgaagaaaataagcaacgtgtcaaaagtatgattaacattttacagctcgtggccccaggaacaccactgcgcgaagggatagataatgtacttcgcgcacaaacggg
gggactaattgttcttgggtataatgagcagattaaaagcattgttgatggtggttttcatattaattgcgcattctctcctgctagtttatatgaattagcaaaaatggatggagcactt
attttaaatgaaactggaagtaaaattttaattgcaaacgcacagttagttccagattcatctattgattctattgaaacaggtatgcgtcaccgaacagcagagcgtgtagcaaag
cagacgggcagccttgttgtggccatttcacaaagacgtaatgtaattacgctatatcaagggaacttacgttatacattaaaagatataggtgtcattttaacgaaggcaaatcaa
gcaattcaaacgctagaaaaatataaggctgtatggaatgacggcattacgaatttgggtattctagaatttgaagaggtcgttacaatgtccgaggtggttcacgttttacatagt
gttgaaatggtgctgcgtattaaaaatgaaatattgagctatattcatgagttaggaacagaaggtaggttaattcgtttacagcttacagaattactagctgatttagaggcagag
gcagcattgttaattaaagattatcaccaggagaaaacacaagaccatcatcaaatcttgaaaaagttacaagatcttgcaaatacacaacttttagaggatagtgatttagttaaa
ttgcttggctatccaggacaaacaagtttagaagaaagtgtgacacctagagggtaccgaatcacaagcaagatttctcgtgttccgccacttattatcgagaatttaattaatcga
tttaaaacattgcaaggtgtttgccgagcaactattaatgaattggatgatgtggaaggaattggagaagtgagagcgaagaaaatacgagaaggtctaaaaagaattcaaga
gcatctctatatgagtagacacaat**TAA**
