# Supplementary material for: Functional analysis of the sporulation-specific diadenylate cyclase CdaS in Bacillus thuringiensis
Source: Front Microbiol. 2015 Sep 14;6:908. doi: 10.3389/fmicb.2015.00908 (PMC4568413; doi:10.3389/fmicb.2015.00908)
Supplement: Supplementary file 11 [file Image9.PDF]

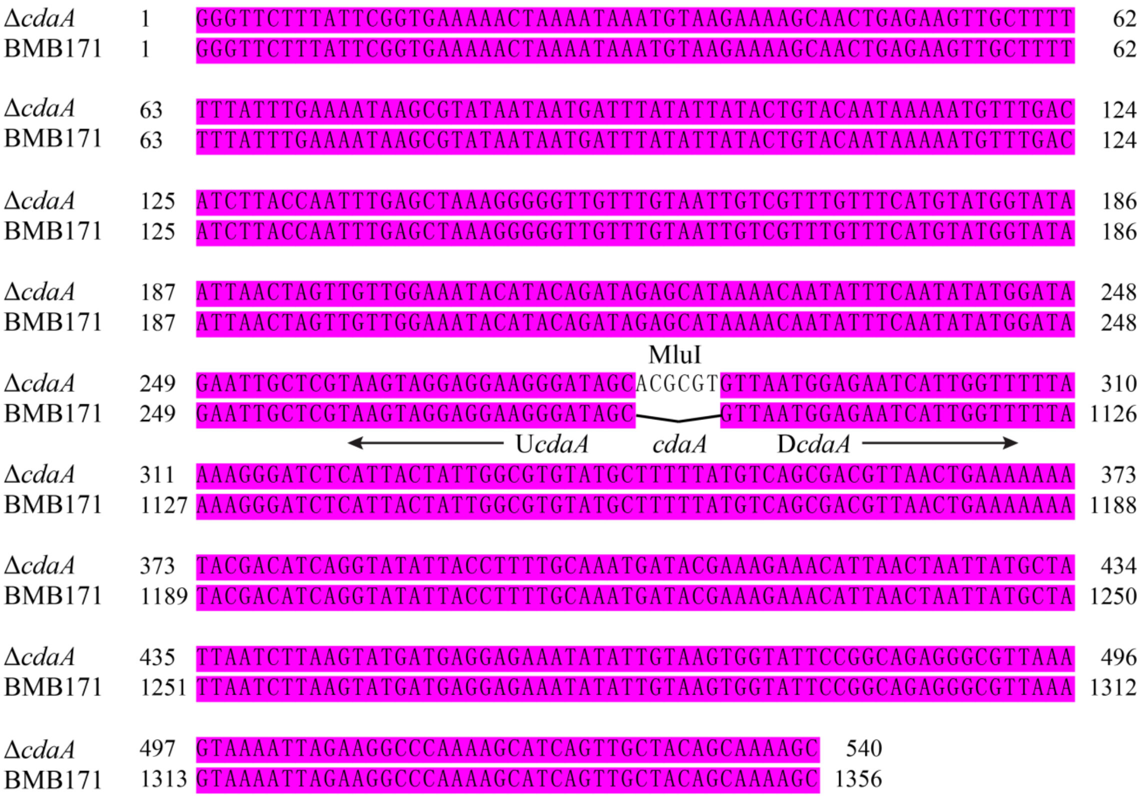

Figure S9. Verification of Δ*cdaA* by sequencing. Sequence alignment of PCR products amplified from the Δ*cdaA* genomic DNA and the BMB171 genomic DNA using primer pair *UcdaA* F/*DcdaA* R. The PCR products (about upstream 280 bp and downstream 250 bp sequences of *cdaA*) were shown. The restriction site of MluI ACGCGT residues in the *cdaA* locus of the BMB171 chromosome (NC_014171, GI: 296500838). What is missing is the *cdaA* gene complete sequence (*BMB171_C0149*, PID: 296500987, in the region 161214..162035 of NC_014171), and it is also listed as follows:

ATGccttttgaagatacgaccattttgaaatatcttagtacggcattagatattgccattgtatggtttattatatataagctaattcttataatccgagggacgaaagctgttcaact
tttaaaagggattacagttattatcgtcgttaagatgatcagtattttccttgaattgcgtacgctatcttggctaactgaacaagtattaacgtggggatttttagccgttattattatctt
tcagccagaattgcgaagagcacttgagcagctagggcgcgggagtttattttcgcgtgttggaacgcatgaggatgatgaacctgaaatggttgcaacagcgatagcaaaa
gcaaccgaatatatggggaaacgtagaattggtgcattaattactttgtcaaaagagaccggtatgggtgattatgtggaaacgggtattccgcttaatgcaaacgtatcatcgg
aattacttattaatatttttattccaaatacacctcttcatgacggagcggtaattatgcaaggaagtacaattaaagcagcagcatgctatcttccattatcagaaagtccgtttatttc
taaagaattaggaactagacatcgtgctgcaatgggagttagtgaagttactgatagtattacagtagttgtgtctgaagaaactggtcaaatttctttaacgaaaaatggtaagtt
gcatcgtgatttgaagacagaacagctgaaagatatgttattagctgaatttagtggaaacgaaaaaacgacttcttcgtctttatggaattggaggagaaagcgtcatgga**TA**
A
