# Supplementary material for: Functional analysis of the sporulation-specific diadenylate cyclase CdaS in Bacillus thuringiensis
Source: Front Microbiol. 2015 Sep 14;6:908. doi: 10.3389/fmicb.2015.00908 (PMC4568413; doi:10.3389/fmicb.2015.00908)
Supplement: Supplementary file 12 [file Image10.PDF]

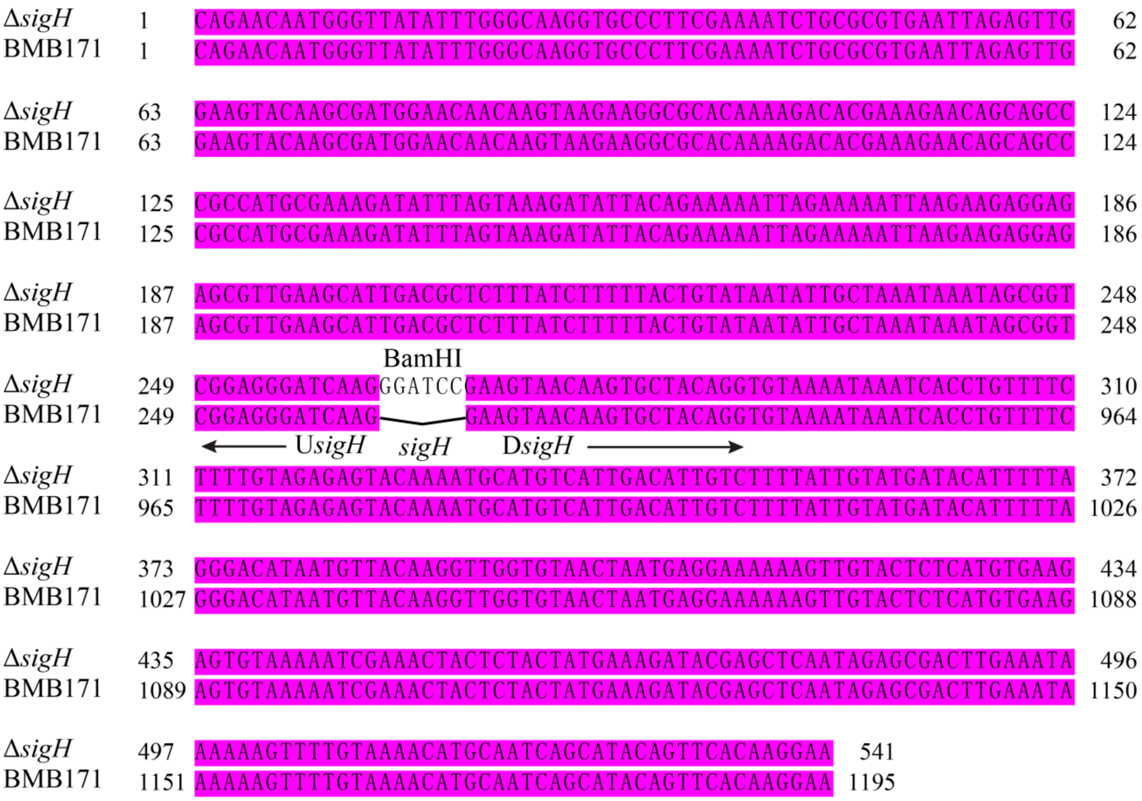

Figure S10. Verification of Δ*sigH* by sequencing. Sequence alignment of PCR products amplified from the Δ*sigH* genomic DNA and the BMB171 genomic DNA using primer pair *UsigH* F/*DsigH* R. The PCR products (about upstream 260 bp and downstream 270 bp sequences of *sigH*) were shown. The restriction site of BamHI GGATCC residues in the *sigH* locus of the BMB171 chromosome (NC_014171, GI: 296500838). What is missing is the *sigH* gene complete sequence (*BMB171_C0090*, PID: 296500928, in the region 109860..110519 of NC_014171), and it is also listed as follows:

GTGgaagcaggcttcgtaagtataggcgacgttacatttcgtgatttagaggatgaggcaatcgttgagttagttcgaaaaggtaatactgacgctctagaatatttaattcac
aagtataagaactttgttcgtgcgaaatcaagatcttactttttagtgggtgccgatcgagaagacattgttcaagaaggtatgatagggttgtttaaagcaattcgtgattataaag
aggacaagctgtcttcattcaaagcatttgctgaactatgtatcactcgacaaattattaccgctattaaaacggcaacaagacaaaaacatattcctttaaattcgtatgtgtcttta
gataagccgatttacgatgaggaatctgatcgaacgttattggatgttatttctgaagcgaaggtgactgatcctgaagagatgatcattagccaggaagaatatacagacatag
aatcaaaaatatctgaattattaagcgatttagaaaggaaagtgctttctttatatttagatggtcgttcttatcaagagatttcagaacagttaaacaggcatgtgaaatctattgata
acgctttacaaagagtaaagagaaaattggaacgatatatggaaatgagagaaagtacaactttaaattca**TAA**
