# Supplementary material for: Functional analysis of the sporulation-specific diadenylate cyclase CdaS in Bacillus thuringiensis
Source: Front Microbiol. 2015 Sep 14;6:908. doi: 10.3389/fmicb.2015.00908 (PMC4568413; doi:10.3389/fmicb.2015.00908)
Supplement: Supplementary file 13 [file Image11.PDF]

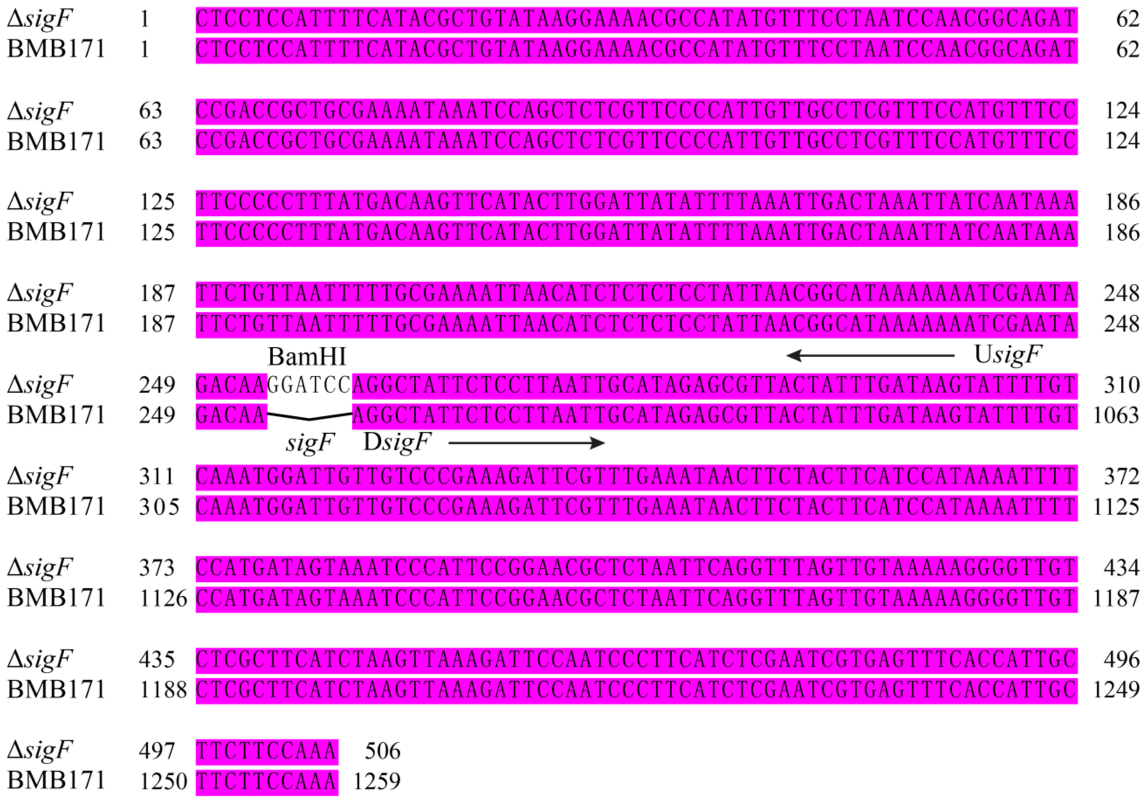

Figure S11. Verification of Δ*sigF* by sequencing. Sequence alignment of PCR products amplified from the Δ*sigF* genomic DNA and the BMB171 genomic DNA using primer pair *UsigF* F/*DsigF* R. The PCR products (about upstream 250 bp and downstream 250 bp sequences of *sigF*) were shown. The restriction site of BamHI GGATCC residues in the *sigF* locus of the BMB171 chromosome (NC_014171, GI: 296500838). What is missing is the *sigF* gene complete sequence (*BMB171_C3737*, PID: 296504567, in the region 3965181..3965939 of NC_014171), and it is also listed as follows:

ATGgacatagaggtcaaaaatgagaagaagaaacctcagttaaaggaccacgagctaaaagcgttaattcaaaaaagtcaagatggagatcaacaagcgagagatacaa tcgttcaaagtaatatgcgcctcgtttggtcggttgtacagcggtttcttaatcgaggatacgaaccagacgacctatttcaaattggatgtattgggctcttgaaatcggtagataa atttgatttatctttcgacgtgaaattttcaacatatgcagttccaatgattattggtgaaatacaacgtttcttacgcgatgatggatcagtgaaagttagtaggtctttaaaagaaac aggaaacaaaattcgaaagatgagagatgagctttcgaaagaattcggaagggctccaacgattaatgaagtggcagaggcactagaactaacgccagaggaagttgttcta gcacaagaagcgagccgggccccttcatctatacatgaaactgtatatgaaaacgatggagatccaatcactattttagatcaaattgcagatcaatctgaaacgaaatggttcg ataaaattgctttaaaagaagcgattagagaactagatgaacgagagcgcttaattgtatacttgcgttactataaagatcaaacacagtcagaagtagccgagcgcataggcat ttcgcaagtacaagtttcaagacttgaaaagaaaatattaaaacagatgaaagatcgaatagacgaa**TAA**
