# Supplementary material for: Functional analysis of the sporulation-specific diadenylate cyclase CdaS in Bacillus thuringiensis
Source: Front Microbiol. 2015 Sep 14;6:908. doi: 10.3389/fmicb.2015.00908 (PMC4568413; doi:10.3389/fmicb.2015.00908)
Supplement: Supplementary file 14 [file Image12.PDF]

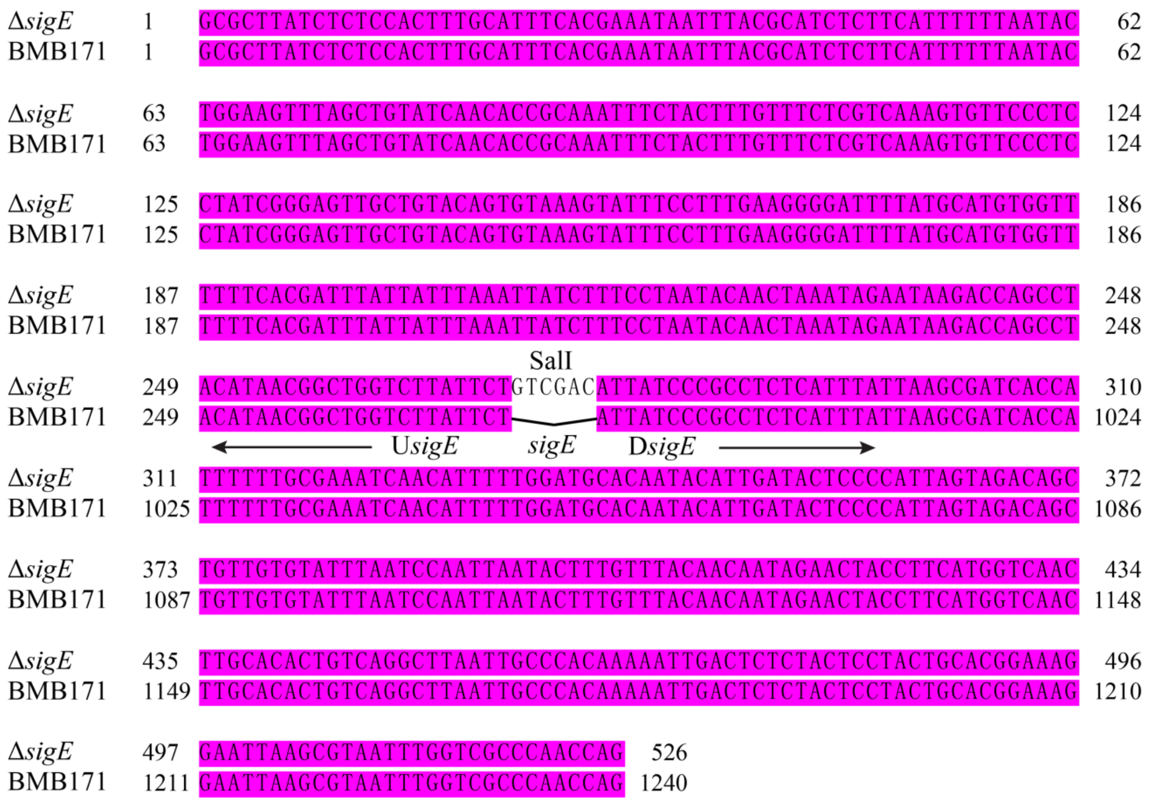


**Figure S12. Verification of Δ*sigE* by sequencing.** Sequence alignment of PCR products amplified from the Δ*sigE* genomic DNA and the BMB171 genomic DNA using primer pair *UsigE* F/*DsigE* R. The PCR products (about upstream 270 bp and downstream 250 bp sequences of *sigE*) were shown. The restriction site of SalI GTCGAC residues in the *sigE* locus of the BMB171 chromosome (NC_014171, GI: 296500838). What is missing is the *sigE* gene complete sequence (*BMB171_C3568*, PID: 296504398, in the region 3806562..3807281 of NC_014171), and it is also listed as follows:

**ATG**atgaaattaaaattttatttagtatacctttggtataaagtattgctgaaattaggaattaagaccgatgaaatttattatattggtggaagtgaagcgttgccaccaccgttaa
caaaagaagaagaggaagttcttttgaataaattgccaaaaggagatcaggcagcaaggtcattacttattgaacgtaacttaaggctcgttgtatatatagcaagaaagtttgaa
aatacagggataaatattgaagatttgattagtataggaacaatcggccttattaaagcggtaaatacatttaatccagaaaagaaaataaaattagcaacatatgcatcgcgttgt
atagaaaatgaaattttaatgcatttacgtcgaaataacaaaaatcgttcggaagtttcttttgatgaaccactaaacattgattgggatgggaatgaactgttattgtctgacgtgtta
ggtacagatgatgatattattacaaaagatttagaagctactgtagatcgtcaccttttaatgaaagcattacaccaattaaatgatcgtgaaaaacaaattatggaacttcggtttg
ggcttgctggaggagaggaaaagacgcaaaaagatgtggctgatatgcttgggatttcacagtcatacatttcgcgtttagaaaaaagaattataaaaagattacgaaaagaat
ttaataaaatggtg**TAA**
