# Supplementary material for: Functional analysis of the sporulation-specific diadenylate cyclase CdaS in Bacillus thuringiensis
Source: Front Microbiol. 2015 Sep 14;6:908. doi: 10.3389/fmicb.2015.00908 (PMC4568413; doi:10.3389/fmicb.2015.00908)
Supplement: Supplementary file 15 [file Image13.PDF]

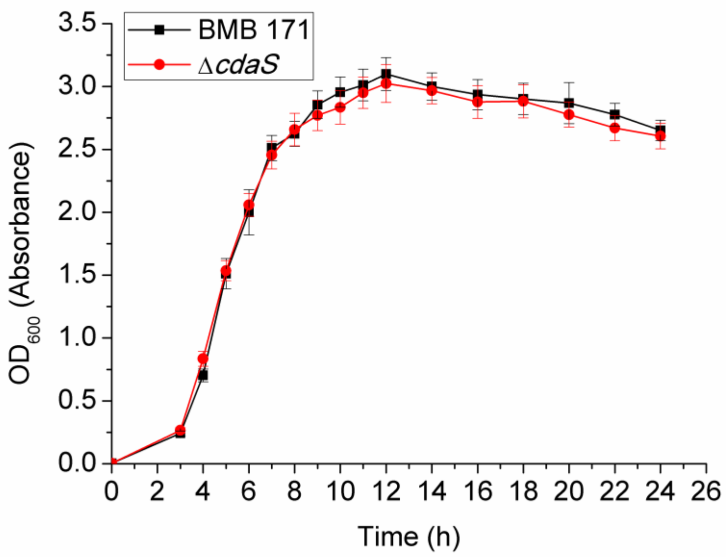

Figure S13. Growth curves of strains BMB171 and Δ*cdaS* in GYS medium. The y-axis presents the average optical densities of triplicate bacterial cultures at 600 nm at each time point. Data are averages of three independent experiments (error bars: SEM).
