# Supplementary material for: Functional analysis of the sporulation-specific diadenylate cyclase CdaS in Bacillus thuringiensis
Source: Front Microbiol. 2015 Sep 14;6:908. doi: 10.3389/fmicb.2015.00908 (PMC4568413; doi:10.3389/fmicb.2015.00908)
Supplement: Supplementary file 16 [file Image14.PDF]

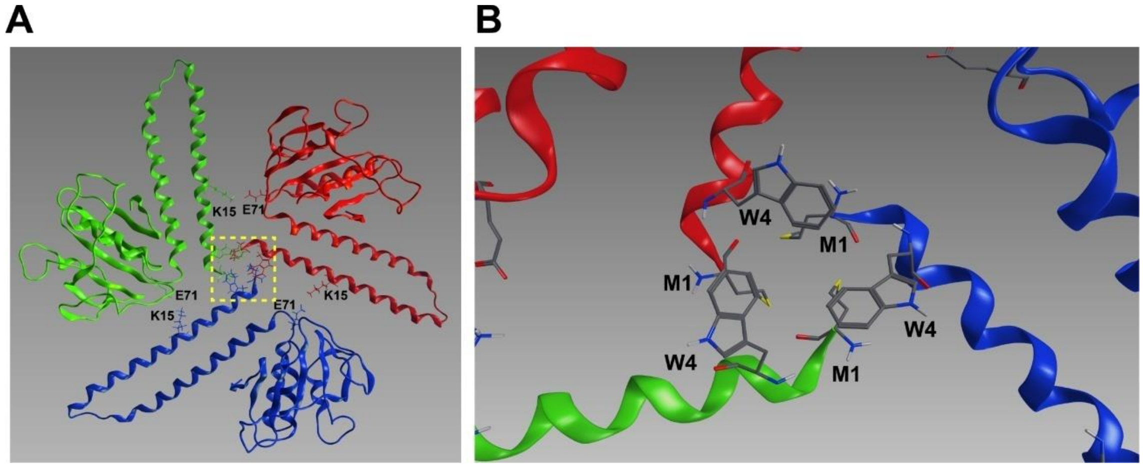


**Figure S14. Proposed important residues that participate in the trimerization of *B. thuringiensis* CdaS.** (A) Ribbon diagram of CdaS homotrimer, and important residues are displayed. The sequence of *B. thuringiensis* CdaS is entirely consistent with *B. cereus* CdaS (PDB code 2FB5), so the homotrimer structure of *B. thuringiensis* CdaS was obtained just delete the redundant three residues in front of Met4 (M4) form 2FB5. The three individual monomers are colored green, blue and red. The three monomers are attracted flabbily to one another by electrostatic interaction between K15 (positively charged) and E71 (negatively charged), and then hanged tightly to each through hydrophobic interactions which are highlighted by yellow dashed box (B). The most important residues for core hydrophobic interactions are M1 and W4 in each monomer, this interaction formed as the hinge to stabilize trimerization. However, G2 and E8 also involve in the hydrophobic interactions. (A) and (B) were drawn using MOE (MOE: The Molecular Operating Environment. Version 2012.10. Chemical Computing Group Inc., 1255 University Street, Suite 1600; Montreal, Quebec, Canada H3B 3X3).
