# Supplementary material for: Functional analysis of the sporulation-specific diadenylate cyclase CdaS in Bacillus thuringiensis
Source: Front Microbiol. 2015 Sep 14;6:908. doi: 10.3389/fmicb.2015.00908 (PMC4568413; doi:10.3389/fmicb.2015.00908)
Supplement: Supplementary file 17 [file Image15.PDF]

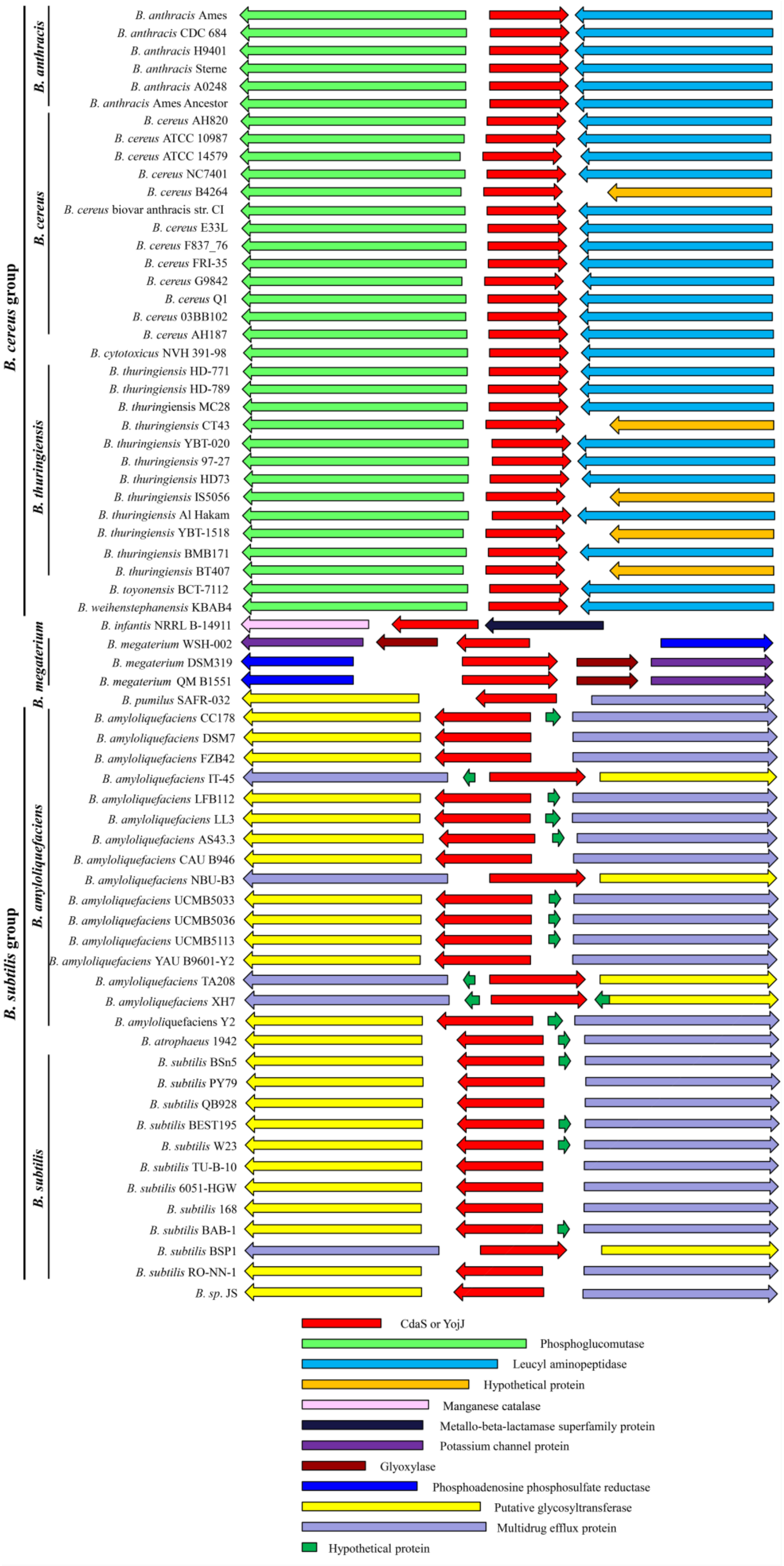

Figure S15. The context of *cdaS* (or *yojJ*) gene in genus *Bacillus*. The analysis involved 68 strains that harbor *cdaS* gene. The conversed genes are showed with the same color, and the functions of these genes are given in the bottom.
